# The effectiveness of clinical networks in improving quality of care and patient outcomes: a systematic review of quantitative and qualitative studies

**DOI:** 10.1186/s12913-016-1615-z

**Published:** 2016-08-08

**Authors:** Bernadette Bea Brown, Cyra Patel, Elizabeth McInnes, Nicholas Mays, Jane Young, Mary Haines

**Affiliations:** 1 Sax Institute, PO Box K617, Haymarket, NSW 1240 Australia; 2 Nursing Research Institute, St Vincent’s Health Australia (Sydney) & Australian Catholic University, School of Nursing, Midwifery and Paramedicine, Darlinghurst, NSW 2010 Australia; 3 Department of Health Services Research and Policy, London School of Hygiene and Tropical Medicine, 15-17 Tavistock Place, London, WC1H 9SH UK; 4 School of Public Health, University of Sydney, Sydney, NSW 2006 Australia

**Keywords:** Clinical networks, Delivery of healthcare, Health system planning, Organisation of healthcare, Health services, Implementation science, Quality improvement

## Abstract

**Background:**

Reorganisation of healthcare services into networks of clinical experts is increasing as a strategy to promote the uptake of evidence based practice and to improve patient care. This is reflected in significant financial investment in clinical networks. However, there is still some question as to whether clinical networks are effective vehicles for quality improvement. The aim of this systematic review was to ascertain the effectiveness of clinical networks and identify how successful networks improve quality of care and patient outcomes.

**Methods:**

A systematic search was undertaken in accordance with the PRISMA approach in Medline, Embase, CINAHL and PubMed for relevant papers between 1 January 1996 and 30 September 2014. Established protocols were used separately to examine and assess the evidence from quantitative and qualitative primary studies and then integrate findings.

**Results:**

A total of 22 eligible studies (9 quantitative; 13 qualitative) were included. Of the quantitative studies, seven focused on improving quality of care and two focused on improving patient outcomes. Quantitative studies were limited by a lack of rigorous experimental design. The evidence indicates that clinical networks can be effective vehicles for quality improvement in service delivery and patient outcomes across a range of clinical disciplines. However, there was variability in the networks’ ability to make meaningful network- or system-wide change in more complex processes such as those requiring intensive professional education or more comprehensive redesign of care pathways. Findings from qualitative studies indicated networks that had a positive impact on quality of care and patients outcomes were those that had adequate resources, credible leadership and efficient management coupled with effective communication strategies and collaborative trusting relationships.

**Conclusions:**

There is evidence that clinical networks can improve the delivery of healthcare though there are few high quality quantitative studies of their effectiveness. Our findings can provide policymakers with some insight into how to successfully plan and implement clinical networks by ensuring strong clinical leadership, an inclusive organisational culture, adequate resourcing and localised decision-making authority.

**Electronic supplementary material:**

The online version of this article (doi:10.1186/s12913-016-1615-z) contains supplementary material, which is available to authorized users.

## Background

Networks of clinical experts are increasingly being established as a strategy to promote the uptake of evidence based practice and drive improvements in standards of patient care. These clinical networks are argued to represent a shift away from hierarchical, bureaucratic organisation of healthcare services to one which engages clinicians more in the development of improved models of care, integration of services and multidisciplinary collaboration [1, 2]. Broadly, clinical networks provide a structure for clinicians to work more closely across institutional and professional boundaries, and allow for continuous working relationships and flow of knowledge about best practice between individuals and organisations, thereby improving the quality of and access to care for patients, including those who require coordination of care across a range of settings. With this shared aim, clinical networks have been established in the United Kingdom (UK) [3–5], other parts of Europe [6, 7], Australia [1, 8–10], Canada [11], and the United States (US) [12].

The use of networks to reduce fragmentation and increase efficient and seamless integration of service delivery is well established in other public services [13, 14]. There has already been significant financial investment. For example, in the UK NHS England allocated £42 million in the 2013/2014 financial year (approximately $27.7 m USD) to the establishment of strategic clinical networks to strengthen the existing less formalised clinical networks [15, 16]. In Australia, $58 million AUD (approximately $48.7 m USD) was allocated in the 2010/11 Budget for the establishment of Lead Clinicians’ Groups in Local Hospital Networks [17]. However, the question remains: does the planning and delivery of services through clinical networks improve quality of care?

The term “clinical network” has been used to describe many variants of networks [2, 18] (Table 1). For this review, we excluded information networks, which are largely soft networks whereby members list themselves in an electronic directory to receive information and resources. Studies of fully integrated service delivery systems were also excluded because they are very contextually specific with overarching administrative structures through which networked services are delivered (e.g. Kaiser Permanente or the Veterans’ Health Administration in the US). Additionally, ‘communities of practice’ were excluded because there has been a systematic review published which assessed the evidence of whether they improved the uptake of best practices and mentoring of new practitioners in the health sector [19]. That review identified 13 primary studies, none of which met the eligibility criteria for quantitative analysis to evaluate effectiveness. Consequently, the effectiveness of ‘communities of practice’ in the healthcare sector remains unknown.

**Table 1:** Typology of clinical networks

	Community of practice	Information network	Clinical network (non-managed)	Clinical network (managed)	Integrated service delivery
Definition	Groups of people who share a concern or passion for something they do and learn how to do it better as they interact regularly. Communities of practice are characterised by voluntary and transitory memberships without a hierarchical structure.	Soft networks are largely referral systems whereby members list themselves in an electronic directory to receive information and resources.	Groups of voluntary experts who work together on common concerns to develop solutions that involve transcending traditional boundaries. These networks are characterised by a hierarchical structure with governance arrangements. These tend to be organised by clinical discipline.	Groups of clinicians who deliver services across boundaries between healthcare professions and the different sectors of the health system. These tend to be organised by clinical discipline.	Networks made up of healthcare organisations as well as individuals within them with an overarching administrative structure with a focus on integration and coordination of clinical services. These tend to be organised by geographical region.
Membership	Individuals Flexible and unrestricted	Individuals Flexible and unrestricted	Individuals Flexible and voluntary	Individuals and healthcare organisations Formal	Healthcare organisations Contractual arrangements about service delivery
Governance and management	Non-hierarchical and informal “Bottom up”	Non-hierarchical and informal “Bottom up”	Semi-hierarchical “Bottom up”	Hierarchical “Mix of bottom up and top down”	Hierarchical “Top down”
Overlap with other typology	Enclave^a^	Enclave	Individualistic	Individualistic	Hierarchical
Example	Canadian Health Services Research Foundation - The Executive Training for Research Application (EXTRA) program alumni community of practice, Canada http://www.cfhi-fcass.ca/sf-docs/default-source/extra/cfhi-extra_brochure-2015-e.pdf	NHS UK – CHAIN: Contact, Help, Advice and Information Network, UK http://chain.ulcc.ac.uk/chain/index.html	NSW Agency for Clinical Innovation’s networks, Australia http://www.aci.health.nsw.gov.au/	NHS National Services Division Scotland Managed Clinical Networks, UK http://www.nsd.scot.nhs.uk/services/nmcn/index.html	Veterans Integrated Service Networks, Veterans’ Health Administration, US http://www2.va.gov/directory/guide/division_flsh.asp?dnum=1
Included in this review	Not included	Not included	Included	Included	Not included

^a^Enclave is defined where members are individuals rather than organisations whose participation is voluntary and often transient

Previous systematic reviews [2, 19] of other models of clinical networks were not able to draw conclusions because of limited and poor quality research. This is a fairly common conclusion for reviews of newly established, innovative healthcare structures, processes and systems [20–22]. A large-scale systematic review of clinical networks published in 2004 described models and functions of networks across multiple public service sectors [2]. That review had a broad focus in order to derive implications for management, governance, leadership and policy of networks in health and social care. In relation to healthcare, this review concluded that there was no evidence of how effective networks were in improving patient care. A more recent review focused on the structure of social networks of health professionals concluded, “cohesive and collaborative health professional networks can facilitate the coordination of care and contribute to improving quality and safety of care” [23].

The current review focuses on managed and non-managed clinical networks, defined as voluntary clinician groupings that aim to improve clinical care and service delivery using a collegial approach to identify and implement a range of quality improvement strategies [8] (see Table 1 for further definitions). The primary aim was to investigate the effectiveness of these clinical networks to improve: a) quality of care (for example, increased uptake of evidence based practice through development and dissemination of clinical practice guidelines and protocols, or care pathway redesign); and b) patient outcomes (based on objective outcome measures). A subsidiary aim was to identify how clinical networks achieved their impacts: evidence of impact on quality of care and patient outcomes from quantitative studies was supplemented with findings of qualitative research to aid interpretation of results and facilitate understanding of the process of network implementation, network structure, the ways in which networks have been used to improve knowledge sharing and coordination of services, and key features necessary for success.

## Methods

The review was conducted in accordance with the Preferred Reporting Items for Systematic Reviews and Meta-Analyses (PRISMA) approach [24] (Additional file 1 contains a detailed description of the methods). Review methods were adapted for a mixed methods systematic review using the framework outlined by Thomas and colleagues [25, 26] which allows independent syntheses of quantitative and qualitative studies followed by integration of findings. Given the lack of high quality evidence from randomised controlled trials, we adopted a pragmatic approach, examining all available evidence, from primary observational studies, and assessing quality within this lower level of the evidence hierarchy using established protocols.

### Search strategy

**Fig. 1:**
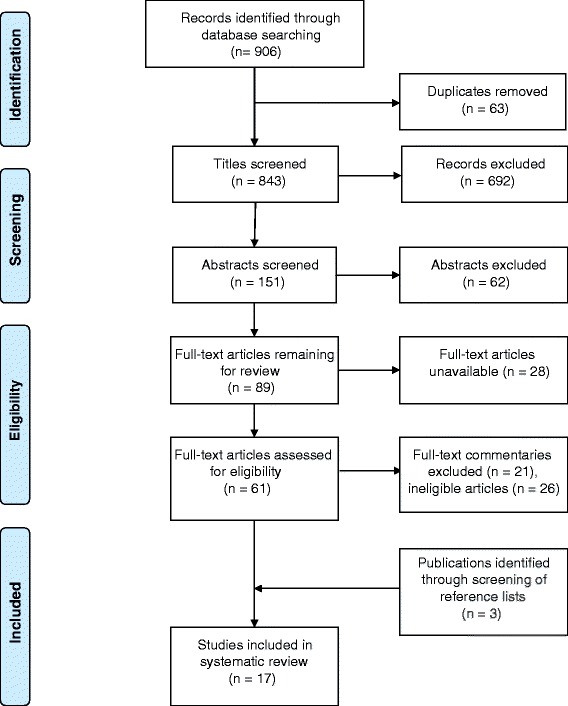
PRISMA Flow Diagram – Initial search 1996–2010

**Fig. 2:**
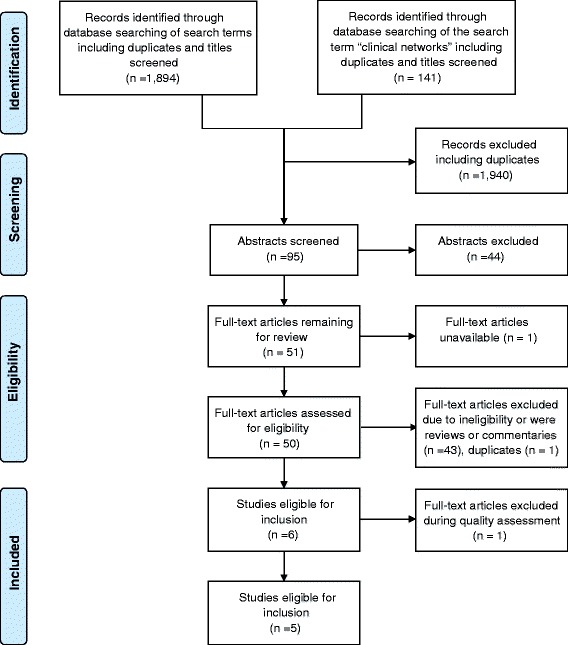
PRISMA Flow Diagram – Updated search 2011-September 2014

The search was conducted in two stages (Figs. 1 and 2). Papers published between 1996 and 2010 were identified by searching the electronic databases Medline, Embase and CINAHL, as well as by snowballing. The search was updated later in PubMed and CINAHL to include publications between 1 January 2011 and 30 September 2014.

Articles were included if the primary focus was on clinical networks as defined in Table 1 (see Additional file 1 for full eligibility criteria). The reviewers independently reviewed abstracts and selected full text articles to confirm whether the publication should be included in the analysis. Discrepancies were resolved through discussion and consensus.

With 17 articles from the initial search and 5 from the updated search, a total of 9 quantitative and 13 qualitative eligible studies were identified for the search period 1 January 1996 to 30 September 2014.

### Quality and assessment of risk bias

Quality assessments of quantitative and qualitative studies were conducted separately [27, 28]. Two review authors (BB, CP) independently assessed the risk of bias of each study; discrepancies were resolved by consensus with a third author (MH) as needed. Studies were grouped into three categories (high, medium and low). For the quantitative studies, the reviewers agreed that observational articles would not be given a “high” quality rating even when bias was minimised in the study due to the difficulty in controlling confounding and attributing causality when using an observational design for effectiveness studies. Following discussion, there was 100 % agreement on the quality assessment rating of the included articles between the three researchers (see Table 2). Quality ratings were used descriptively to assess the strength of evidence.

**Table 2:** Summary of included quantitative articles

Authors	Country	Type of network	Theme	Study design	Quality rating^a^
Gale et al. 2012 [3]	UK	Managed clinical network for neonatal services	Improving quality of care	Observational – before and after	Moderate
Greene et al. 2009 [31]	UK	Tayside Diabetes Managed Clinical Network	Improving quality of care	Observational – cross-sectional	Moderate
Hamilton et al. 2005 [4]	Scotland	Managed clinical network for cardiac services	Improving quality of care	Quasi-experimental – interrupted time series	Moderate
McClellan et al. 1999 [33]	USA	End Stage Renal Disease Networks	Improving patient outcomes	Observational – before and after	Low
McCullough et al. 2014 [30]	Scotland	Scottish Sarcoma Managed Clinical Network	Improving quality of care	Observational – retrospective before and after	Low
Ray-Coquard et al. 2002 [6]	France	Regional cancer network of hospitals	Improving quality of care	Quasi-experimental – controlled before and after	Moderate
Ray-Coquard et al. 2005 [7]	France	Regional cancer network of hospitals	Improving quality of care	Observational – before and after	Moderate
Spence & Henderson-Smart 2010 [32]	Australia	Australian and New Zealand Neonatal Network	Improving quality of care	Observational – before and after	Low
Tideman et al. 2014 [34]	Australia	Integrated cardiac support network	Improving patient outcomes	Observational – retrospective before and after	Moderate

^a^Quality rating definitions are as follows

• High quality – design and conduct of study address risk of bias, appropriate measurement of outcomes, appropriate statistical and analytical methods, low drop-out rates, adequate reporting

• Moderate quality – do not meet all criteria for a rating of good quality but no flaw is likely to cause major bias, some missing information

• Low quality – significant biases including inappropriate design, conduct, analysis or reporting, large amounts of missing information, discrepancies in reporting

### Data extraction and synthesis

Data from quantitative and qualitative papers were extracted into separate data extraction tables (Additional file 2). Quantitative papers were categorised independently by two reviewers (BB, CP) according to the focus of the study: 1. quality of care; or 2. patient outcomes (Table 2). Due to the heterogeneity of the included quantitative studies and their outcomes, results were reported in narrative form. Qualitative methods were used to thematically analyse and synthesise textual data extracted from the qualitative studies [29]. The two authors independently identified the focus of the qualitative papers and categorised them under four agreed themes: 1. features and outcomes of effective networks; 2. network implementation; 3. organisational structure; or 4. organisational learning and knowledge (Table 4). Results from the quantitative narrative analysis were then integrated with the qualitative synthesis in the discussion to identify recurrent themes and explain how successful networks achieved their outcomes.

## Results

Additional file 2 presents an overview of the 22 studies including details of context, sample, research aim, study design, methods, outcomes and main results.

### Synthesis of quantitative studies

Table 2 summarises study characteristics and quality ratings. With the exception of one study published in 1999, the remainder (eight) were published after 2000, with four published since 2011. Four studies were undertaken in the UK, two in France, two in Australia and one in the US. The studies involved networks covering diverse clinical specialties including: cancer (three); cardiac services (two); diabetes (one); end stage renal disease (one); and neonatal services (two).

Of the nine included quantitative studies, seven focused on quality of care and two focused on patient outcomes (see Additional file 2 for measures used in each study). Based on our quality assessment criteria, six studies (67) were of moderate quality and three studies (33 %) were of low quality (Table 2). Studies were limited by the use of observational rather than experimental designs (7 of 9).

Four studies assessed the impact of the establishment and reorganisation of healthcare into clinical networks, while five studies assessed the impact of network initiatives. Network initiatives included development and dissemination of clinical practice guidelines and protocols, educational activities (e.g. workshops), clinical audit and provision of feedback, care pathway redesign, facilitation of multidisciplinary team care, patient education, and other interventions to improve clinical care (such as point-of-care reminders and availability of new technology).

#### Effectiveness of clinical networks to improve quality of care

A total of seven studies examined quality of care indicators, all of which achieved significant improvements on some or all indicators. Studies are listed by clinical specialty. Results are summarised in Table 3 and detailed in Additional file 2.

**Table 3:** Summary of findings from quantitative articles

Study	Intervention	Improvement observed?	Summary of findings	Significant results
Gale et al. 2012 [3]	National reorganisation of neonatal services in England into managed clinical neonatal networks to improve access to specialist care for pre-term births	Yes	Improvement in primary outcomes, less success in secondary outcomes	Increase in •Proportion of babies born at 27–28 weeks gestation at hospitals providing the highest volume of specialist care (18 % to 49 %; risk difference 31 %; 95 % CI [28 % to 33 %]; OR: 4.30; 95 % CI [3.83 to 4.82]; *p* < 0.001). •Proportion of babies undergoing acute and late postnatal transfer in England (7 vs. 12 and 18 % vs. 22 %, respectively; *p* < 0.001).
Greene et al. 2009 [31]	Progressive implementation of multiple quality improvement strategies including; guideline development and dissemination; education; clinical audit, feedback and benchmarking; encouragement of multidisciplinary team working; task redesign; and care pathway redesign	Yes	Rapid improvement in simple indicators, slow improvement in complex indicators	Improvements (all *p* < 0.001) in measurement/assessment and recording of: •Glycated haemoglobin •Blood pressure •Cholesterol •Smoking status •Creatinine •Foot vascular and neurological status •Retinal screening
Hamilton et al. 2005 [4]	Establishment of a managed clinical network for cardiac services in a predominantly rural area to improve patient care	Some	Improvement in 11 indicators (2 significant, 9 non-significant); no improvement in 5 indicators	Improvement in: •Pain to needle time <90 min; *p* = 0.05 •70 % on beta-blockade at 6 months post myocardial infarction; *p* = 0.05
McClellan et al. 1999 [33]	A multifaceted intervention through a clinical network to improve haemodialysis adequacy	Yes	Improvement in all primary outcomes	•Improvement in Urea Reduction Ratios (URRs) from 63 % to 67 % (*p* < 0.001) •Decrease in the proportion of under-dialysed patients from 56.6 % to 31.7 % (chi-squared for trend, *p* < 0.0001).
McCullough et al. 2014 [30]	Establishment of the Sarcoma Managed Clinical Network to improve the quality of diagnosis, treatment and care of sarcoma patients including facilitating national multidisciplinary discussion of all sarcoma cases, registering case details and provision of care by a multidisciplinary team	Some	Improvement in all primary outcomes, but decline in some secondary outcomes	•Decreased time interval from referral to initial assessment by the service from median 19.5 days to 10 days. •Increase in proportion of patients undergoing investigation with a MRI scan prior to excision of the sarcoma from 67 % to 86 % a (*p* = 0.0009). •Increase in proportion of patients undergoing appropriate biopsy from 57 % to 79 % (*p* = 0.006). •Increase in complete resection margins from 48 % to 81 % (*p* < 0.001).
Ray-Coquard et al. 2002 [6]	Implementation of cancer clinical practice guidelines (CPGs) through a regional clinical network	Some	Improvement in compliance to some clinical guidelines but not all	Compliance of overall treatment sequences post-implementation of clinical practice guidelines in Network hospitals: •*Colon cancer* 46 %; 95 % CI [30 % to 54 %] (56 out of 123) vs. 14 %; 95 % CI [7 % to 21 %] (14 out of 103); *p* < 0.001 •*Breast cancer* 36 %; 95 % CI [30 % to 42 %] (126 out of 346) vs. 12 %; 95 % CI [8 % to 16 %] (34 out of 282); *p* < 0.001 Control group: •No significant difference
Ray-Coquard et al. 2005 [7]	Sustained adherence to cancer clinical practice guidelines within a regional clinical network	Yes	Sustained improvement in compliance to clinical guidelines	Compliance of medical decisions with clinical practice guidelines at 3-year follow-up in: Network hospitals: •*Colon cancer* 73 %; 95 % CI [67 % to 79 %] v 56 %; 95 % CI [49 % to 63 %] respectively; *p* = 0.003 •*Breast cancer* 36 %; 95 % CI [31 % to 41 %] v 40 %; 95 % CI [35 % to 44 %] respectively; *p* = 0.24 Control group: •*Colon cancer* 67 % 95 % CI [58 %–76 %] v 38 %; 95 % CI [29 %–47 %] respectively; *p* = 0.001 •*Breast cancer* 4 %; 95 % CI [1 %–7 %] v 7 %; 95 % CI [3 %–11 %] respectively; *p* = 0.19.
Spence & Henderson-Smart 2010 [32]	A multifaceted intervention through a clinical network to support practice change and close the evidence-practice gap for newborn pain management	Some	Improvement in several primary outcomes with a process for sustainability established for goals not achieved	Increase in: •Percentage of infants receiving sucrose for procedural pain (41 % to 61 %; *p* < 0.005) •Staff awareness of a clinical practice guideline for the management of newborn pain (61 % to 86 %; chi square = 73.8, d.f. 1, *p* = 0.000) •Family awareness of infant pain and strategies to manage the pain (19 % to 48 %; chi square = 52.3, d.f. 1, *p* = 0.000).
Tideman et al. 2014 [34]	Establishment of a regionalised integrated Cardiovascular clinical network to reduce mortality in patients with acute myocardial infarction in hospitals in a rural setting	Yes	Improvement in all primary outcomes	•Decrease in 30-day mortality among patients presenting to hospitals integrated into the clinical network (13.93 % vs 8.92 %; *p* < 0.001). •22 % relative odds reduction in 30-day mortality compared with patients presenting to rural centres outside the clinical network (OR, 0.78; 95 % CI, 0.65–0.93; *p* = 0.007). •Increased rate of transfer of patients to metropolitan hospitals (before ICCNet, 1102/2419 (45.56 %) vs. after ICCNet, 2100/3211 (65.4 %); *p* < 0.001).

- *Cancer*
  Three observational studies reported significant improvements on quality of care indicators related to previous provision of cancer services. In a controlled before and after study, Ray-Coquard et al. [6] reported a significant increase in the observed compliance rate for overall treatment sequences for breast (36 v 12) and colon (46 % v 14 %) cancer (both *p* < 0.001) post-implementation of clinical practice guidelines established and disseminated by a regional cancer network for hospitals in the network. In the control group of non-network hospitals, there was no significant difference in the observed compliance rate pre-and post-implementation. In a three-year follow up repeated controlled before and after study of the same network clinical practice guideline implementation initiative, Ray-Coquard et al. [7] observed that compliance of medical decisions with clinical practice guidelines was significantly higher in network hospitals at follow up for colon cancer (73 % v 56 %; *p* = 0.003) and similar for the two periods for breast cancer (36 % v 40 %; *p* = 0.24). In the control group, compliance was significantly higher at three-year follow up for colon cancer (67 % v 38 %; *p* = 0.001) and the same for the two periods for breast cancer. While there was improvement in compliance for colon cancer in both networked and non-networked hospitals at three-year follow up, behaviour change was more rapid in the region within the cancer network suggesting that evidence based information was disseminated more expeditiously through the network and improvements were sustained over time.
  In a retrospective observational study, McCullough et al. [30] conducted a cohort analysis of patient records and administrative datasets before and after establishment of the Scottish Sarcoma Managed Clinical Network. More patients were seen by more specialties after establishment of the network and there were significant improvements in the time interval from receipt of referral to initial assessment, the proportion of patients undergoing investigation with a magnetic resonance imaging (MRI) scan prior to excision of the sarcoma (67 to 86; *p* = 0.0009), and the proportion of patients undergoing appropriate biopsy (57 % to 79 %; *p* = 0.006). The rate of complete resection margins also significantly increased.
- *Cardiac services*
  In one quasi-experimental interrupted time series study, Hamilton et al. [4] reported statistically significant improvement in two out of 16 clinical care indicators (pain to needle time <90 min; *p* = 0.05 and 70 % on beta-blockade at 6 months post myocardial infarction; *p* = 0.05) and non-significant improvement in nine others following the set-up of a managed care network for cardiac services in Scotland. Five indicators showed no significant improvement and there was no impact on costs.
- *Diabetes*
  One study [31] retrospectively evaluated the impact of quality improvement initiatives undertaken by the Tayside Diabetes Managed Clinical Network in the UK using data extracted from the regional diabetes register. Simple process indicators such as measuring glycated haemoglobin, blood pressure and cholesterol rapidly improved, while there was slow continuous improvement of others (all significance levels *p* < 0.001). Improvements were greater for type 2 than type 1 diabetes for which three indicators did not change significantly. Significant shifts of care for type 2 diabetes into primary care were achieved.
- *Neonatal Care*
  Two observational before and after studies, one in Australia [32] and one in the UK [3] reported neonatal care outcomes of neonatal care networks. The previously established Australian and New Zealand Neonatal Network [32] drove the implementation of multiple intervention strategies to increase evidence based practice for the treatment of pain in the newborn, resulting in significant improvements across three outcomes including: increased use of a pain assessment tool for ventilated neonates; an increase in the percentage of infants receiving sucrose for procedural pain (41 % to 61 %; *p* < 0.005); and increased staff awareness of a clinical practice guideline for the management of newborn pain (61 % to 86 %; *p* = 0.000). Family awareness of infant pain and strategies to manage pain also increased (19 % to 48 %; *p* = 0.000).
  In the UK, the impact of reorganisation of neonatal specialist care services for high risk pre-term babies into managed clinical networks for neonatal services achieved significant improvements [3]. The proportion of babies born at 27–28 weeks gestation at hospitals providing the highest volume of specialist care increased significantly (18 % to 49 %; *p* < 0.001), as did the proportion of babies undergoing acute and late postnatal transfer (7 v 12 and 18 % v 22 %, respectively; *p* < 0.001). There was no significant reduction in the number of infants from multiple births separated by transfer.

#### Effectiveness of clinical networks to improve patient outcomes

Two observational (one prospective and one retrospective before and after) studies assessed patient outcome measures, both reporting improvements on primary indicators. A US study [33] reported positive effects of a quality improvement intervention on network-specific Urea Reduction Ratios (URRs) driven by the End Stage Renal Disease Network with improvements in URRs during the intervention period (63 % to 67 %; *p* < 0.001) and a decrease in the proportion of under-dialysed patients in the networks (56.6 % to 31.7 %; *p* < 0.001). Successful intervention strategies included audit and feedback coupled with educational interventions, involvement of a diversity of physicians and clinical leaders, and persistence over several years.

In Australia, the regionalised Cardiovascular Clinical Network (ICCNet) was established to improve outcomes of patients with myocardial infarction (MI) in rural settings [34]. Among rural hospitals, 30-day mortality decreased among patients presenting to hospitals integrated into the clinical network (13.93 % before ICCNet vs 8.92 % after ICCNet; *p* < 0.001). After adjustment for temporal improvement in MI outcome, baseline comorbidities and MI characteristics, presentation to an ICCNet hospital was associated with a reduction in 30-day mortality compared with patients presenting to rural centres outside the clinical network. A strong association between network support and increased rate of transfer of patients to metropolitan hospitals was observed (before ICCNet, 1102/2419 [45.56 %] vs. after ICCNet, 2100/3211 [65.4 %]; *p* < 0.001). Increased transfers were associated with a lower total length of stay compared with admissions before implementation of the network.

### Synthesis of qualitative studies

Table 4 summarises key study characteristics and quality ratings. All of the 13 studies were published in 2005 or later. Eight were undertaken in the UK, two in Australia, two in Canada, and one in Sweden. The majority of studies used a case study or comparative case study approach to examine clinical networks. A summary of findings is available in Additional file 2. Nine of the 13 studies were given a high quality rating while four were given a moderate quality rating. Although none were rated low quality, studies were limited by their lack of use of sufficient strategies to establish reliability (e.g. independent coding) or validity of data analysis (e.g. reporting of negative cases).

**Table 4:** Summary of included qualitative articles

Authors	Country	Type of network	Theme	Study design	Quality rating^a^
Addicott 2008 [41]	UK	Managed clinical network for cancer services	Organisational structure	Comparative case study	High
Addicott & Ferlie 2007 [42]	UK	Managed clinical network for cancer services	Organisational structure	Comparative case study	High
Addicott et al. 2007 [43]	UK	Managed clinical network for cancer services	Organisational structure	Comparative case study	High
Addicott et al. 2006 [44]	UK	Managed clinical network for cancer services	Organisational learning and knowledge	Observational, cross-sectional organisational process study	High
Ahgren & Axelsson 2007 [35]	Sweden	‘Chains of care’ (managed clinical networks) for patients having the same illness or symptom	Features and outcomes of effective networks	Cross-sectional embedded multiple-case study	High
Baker & Wright 2006 [36]	UK	Managed clinical network for paediatric liver services	Features and outcomes of effective networks	Appreciative Inquiry methodology (case study)	Moderate
Burnett et al. 2005 [45]	UK	Various managed clinical networks (cancer, coronary heart disease, stroke, mental health)	Organisational learning and knowledge	Qualitative information and knowledge needs analysis (comparative case study)	Moderate
Cunningham et al. 2012 [23]	Australia	Advisory clinical networks – two networks for musculoskeletal health (NSW and WA)	Features and outcomes of effective networks	Longitudinal comparative case study	High
Fleury et al. 2002 [40]	Canada	Mental health integrated service network	Network implementation	Case study and multi-dimensional analytic model	Moderate
Hogard & Ellis 2010 [38]	UK	Managed clinical network for personality disorder	Features and outcomes of effective networks	Evaluation Trident methodology (case study)	Moderate
McInnes et al. 2012 [39]	Australia	Voluntary collegial clinical networks in NSW established by the NSW Agency for Clinical Innovation	Features and outcomes of effective networks	Comparative case study	High
Tolson et al. 2007 [5]	Scotland	Managed clinical network (Palliative Care), linking primary, secondary and tertiary care	Network implementation	Realistic Evaluation methodology (qualitative pilot case study)	High
Touati et al. 2006 [13]	Canada	Managed clinical network (cancer)	Network implementation	Longitudinal qualitative case study	High

^a^Quality rating definitions are as follows

• High quality – those meeting 8 or more criteria

• Medium quality – those meeting between 5 and 7 criteria

• Low quality – those meeting fewer than five criteria

The full list of 11 criteria can be found in Additional file 1

While five articles specifically addressed the features and outcomes of effective networks, there was significant overlap among articles that described network implementation, organisational structure and the role of networks in organisational learning and knowledge, which similarly noted the importance of network leadership, interpersonal relationships, structure and resourcing as factors that contribute to network effectiveness.

#### Features and outcomes of effective networks

Five papers [35–39] identified the characteristics that enabled networks to be effective in implementing changes that improved quality of care and patient outcomes. Facilitators of and barriers to network success are listed in Table 5. Of note, the availability of sufficient resources, a designated project/network leader or coordinator, strong leadership, a culture of inclusivity, and widespread engagement and participation were recurring features of successful networks. Their absence, particularly inadequate resourcing and lack of management and leadership, was noted to hinder networks’ ability to achieve improvement in quality of care outcomes. The studies noted that success was dependent on a combination of these factors being present rather than just a few isolated features. In particular, commitment to a set of shared values and objectives was necessary but insufficient for clinical effectiveness in the absence of other factors [38].

**Table 5:** Features of successful clinical networks – facilitators and barriers

Facilitators of network success	Barriers to network success
Sufficient resources – funding, administration and human (staffing) Availability of information and communication technologies A bottom-up, locally-initiated and driven approach to network implementation, with subsequent formalisation to increase the adoption of new processes A positive, trusting culture where networks are seen as desirable and perceived to be necessary to sharing knowledge, and where there is open and inclusive communication, clinician engagement and widespread genuine stakeholder participation The norms and values of the network are compatible with those of the organisations involved Strong leadership, particularly by clinical leaders and network managers using a facilitative approach Inclusive membership in the network, including representation of patients and other stakeholders Engagement at different levels of the healthcare system Evidence based work plans and projects that address issues identified by network members, particularly gaps in current practice, with goals that are feasible and can be objectively measured Supportive policy environments and links with government agencies	Lack of funding and resources Tension, distrust and competition (particularly over resources) between network members An imbalance of power between network members resulting in competition for resources Poor communication and unwillingness to collaborate Lack of confidence in the ability of network leaders and managers Lack of representation of key stakeholders in certain contexts (e.g. rural and indigenous interests) Poor record keeping and documentation, which made it difficult to measure the impact of network initiatives and track progress A top-down approach of network implementation, or where implementation is mandated, led by external organisations, and/or decision-making powers and responsibilities are maintained by external parties thereby limiting the powers of network members

Outputs of effective networks included the development or reorganisation of service delivery into clear clinical pathways, provision of holistic services, improved working relationships and collaboration within the network, and improved clinical knowledge and skills of network members.

#### Network implementation

Three articles described the process of implementing a clinical network and the key lessons learned from the implementation process [5, 13, 40]. Two of the studies described positive steps towards the implementation of clinical networks [5, 13], while one study described a negative experience [40]. The overarching lesson was that the implementation of a network is extremely complex and requires “considerable time, resources and initiatives at different levels of the healthcare system” [13]. Successful implementation required strong leadership, coordination and a sense of shared values and trust between network members. While vital, clinical leadership alone was insufficient [13]. Trust between network members, whether inter-organisational or inter-professional, was regarded as being vital to the implementation process. Members had to be receptive to the concept of the network. For this, the values of the network had to match the values of the organisation and of the clinicians involved. Power imbalances between institutions in a network were observed to hinder the implementation process, as larger institutions were viewed as “hoarding resources” leaving smaller practices at a disadvantage, resulting in their disengagement [40].

The availability of adequate resources for the network was also essential. This included funding, administration and human resources. The formalisation of processes was seen as a positive step, but only when done under the direction of the clinical teams. Inexperience in change management and unfamiliarity with leading development projects were cited as barriers to implementation [5]. It was essential for network members to have confidence in the expertise and ability of the people leading the changes to the system; where leaders lacked legitimacy and were perceived to lack the required knowledge and expertise, implementation was slow. Having clinical leaders who championed change was essential for buy-in from other clinical staff [5, 13]. Implementation of the network was also unsuccessful when a top-down approach was used, where the network was mandated and led by external organisations rather than having clinicians set priorities and driving the implementation process. Without genuine participation of the physicians involved, implementation was difficult and did not appear to affect practice [40].

One study reported briefly on some of the outcomes of the implementation process which were generally viewed as positive [5]. There were better working relationships between teams, enhanced knowledge, and a greater commitment to the practice of evidence based care. There also appeared to be improved patient outcomes – interviewed patients reported better management of their symptoms and had greater knowledge about how to manage their condition.

#### Organisational structure

Three articles looked at how networks were structured and how network structure affected the ability to function in the local context [41–43]. All three articles referred to a single study of five managed clinical networks for cancer in the UK. Despite attempting to delegate authority to the local level, the organisational structure of the networks maintained decision-making power at a centralised level. Boards had limited strategic influence, with decision making power and budgetary responsibilities remaining with the statutory authorities; only one board was able to have a noteworthy impact due to the seniority of its members [41]. Due to the top-down approach used to set up these networks by the government, the networks achieved limited success in organising and working together effectively, with only one network emerging as a successful anomaly [41]. At all levels, network members in positions of less influence struggled to make an impact. Network Management Teams relied on interpersonal skills to influence members to cooperate, and were unsuccessful in all but one network [42]. At the local level, a few medical staff overwhelmingly dominated decision-making in all networks, often with the intention of acquiring resources and/or accreditation status for their own institutions [42]. An imbalance of power between medical staff meant that those with less power (typically those clinicians within smaller district hospital units as opposed to those working at a major cancer centre) frequently resisted decisions and implementing changes due to a perception that their interests were not taken into consideration [42].

The organisation of the networks also limited their ability to implement knowledge sharing and educational activities [43]. Because power and influence remained centralised and there was strong resistance to any changes being implemented, there was little impact on organisational processes. Only one network, where the Network Management Team was viewed positively and had an open and facilitative approach to implementing changes, was able to implement some education and training activities. The Team was able to successfully leverage pre-existing relationships to build support for and engagement in the network, and adapt interventions to the local context.

#### Organisational learning and knowledge

Two papers [44, 45] focused on organisational learning and the transfer of knowledge within networks. Members of clinical networks identified organisational learning as a desirable outcome that could increase individual knowledge and improve patient outcomes. They recognised that easy access to timely information would enable them to work more efficiently [45]. However not all networks were able to successfully implement educational measures. Those that were successful had adequate resources, good network management, appropriate organisational structure that facilitated inclusive and open participation, enthusiastic network members and a positive learning environment. Networks where educational initiatives were unsuccessful were characterised by organisational structures that impeded knowledge sharing, poor relationships between network members, weak management and the perception of increasing competition among members. Due to the uneven distribution of resources, individuals competed over resources, which fostered distrust and a lack of willingness to collaborate. Several respondents believed education would become more of a priority when structural issues were addressed [44].

## Discussion

### Testing the effectiveness of clinical networks

There is an emerging, albeit limited, body of empirical quantitative research into the effectiveness of clinical networks. Amongst the nine studies included, the majority (seven) focused on improvement in service delivery. Only two reported on clinical networks’ impacts on patient outcomes. None of the quantitative studies were of high quality, and several (3 of 9) were of low quality. All except two used observational study designs; none used a randomised controlled trial. The lack of studies with a rigorous design limits the conclusions that can be drawn. Although the majority (9 of 13) of the qualitative studies were rated “high quality” and their findings complement those of the empirical studies, they were designed to explain how and why networks may have produced or failed to produce a desired outcome and were not designed to determine whether clinical networks could successfully improve health service delivery and patient outcomes, the latter of which is more appropriately addressed by quantitative studies. None of the studies used mixed methods study designs, which would be useful in assessing why a specific network was or was not effective.

The best available empirical evidence indicates that clinical networks can be effective vehicles for quality improvement. Among the studies reviewed, networks were judged to improve quality based on several endpoints relating to both service delivery (such as adherence to clinical guidelines and protocols, development of clear patient pathways, and use of clinical tools) and patient outcomes (such as reduced mortality, improvement in biomarkers, and improved time to treatment). Desirable intermediate outcomes were also reported in both the quantitative and qualitative studies, such as improved knowledge amongst clinical staff and patients, greater clinical collaboration and greater availability of resources. There is some evidence that clinical networks can be effective in engaging clinicians in service redesign and reform [13], and developing and implementing protocols and clinical practice guidelines [6]. Quality improvement programs undertaken by networks largely report significant improvements across several quality of care indicators for a range of clinical disciplines including cancer [6, 7, 30], diabetes [31], and neonatal care [3, 32]. The two studies reporting patient outcome measures similarly demonstrated positive effects of network-specific interventions for end stage renal disease [33] and reorganisation of cardiac services [34]. There is some evidence to demonstrate that improvements can be sustained over time [7, 33].

Although these findings generally indicate that clinician-led networks can improve care, other studies have not reported such consistent results. One study examining the impact of a managed clinical network for cardiac services on patient care found that only two out of sixteen clinical care indicators significantly improved [4]. The authors note that changes were not noticeable until two years after network start up, which was an intensive process. This resonates with the findings of other studies [31, 46], which found simple process measures rapidly improved but that there was slower improvement across more complex measures that required intensive professional education or comprehensive redesign of the care pathway. There was also variability in the ability of networks to make meaningful network- or system-wide change. A qualitative comparative case study of five cancer networks in the UK conducted by Addicott et al. [44] highlighted a great degree of variability in the extent to which networks successfully implemented planned activities and the consequent success of the network. This would suggest that some quality improvements are likely to be incremental and that complex changes may take longer to be successfully embedded into routine care. Therefore, while clinical networks can be effective in improving care, this is not always the case.

### Features of effective networks

Variability in networks’ success in improving healthcare is multifactorial and dependent on the local context. Implementation of a clinical network and its initiatives is a time- and resource-consuming process [4]. Critical factors for success identified across the qualitative studies were strong leadership by clinical leaders and managers, availability of sufficient resources, and involvement of a broad range of people from different healthcare professions to patients and other stakeholders. The importance of leadership was similarly identified in the two quantitative studies [4, 31] that explored facilitators in the establishment of a network and implementation of network quality improvement initiatives. Successful networks and their initiatives were typically driven by a few individual clinical leaders and dedicated managers who were widely respected by their colleagues and deeply committed to the purpose and values of the networks, which may explain why a strictly ‘top-down’ structure and approach to network implementation was less effective. Furthermore, networks without adequate administrative, human and technological resources were less effective. Several qualitative studies reported that lack of a network manager or project coordinator and insufficient administrative and technological support to improve communication, collect relevant data and share educational tools reduced the effectiveness of networks.

Network structure was also perceived to affect success. Networks where decision-making power was decentralised to the local level were more successful, particularly where they were led by highly respected and trusted clinical leaders and managers [35, 39-43]. Several participants in the qualitative studies noted that without an appropriate organisational structure, the networks were unlikely to be able to change organisational processes and implement quality improvement measures. This could partially explain why some networks were able to change simple process measures like ordering additional laboratory tests, but were unsuccessful in changing more complex processes and systems, like clinical pathways, that may have required the support of a strong network structure.

These findings are in agreement with those of two reports that included an examination of what makes an effective managed clinical network. The first of these by Guthrie et al. [47] in the UK identified the following key factors: *inclusiveness* to ensure that all relevant stakeholders are actively engaged with the network; strong credible *leadership* and *effective management* based on negotiation, facilitation and influence; *adequate resourcing* for network coordination; strong two-way *communication strategies* within the network; and collaborative *relationships with wider organisational context* to ensure network priorities are aligned with those of individual network members as well as local, regional and national organisations and agencies. Respondents in that study additionally agreed that ‘networks should start with relatively small, non-contentious issues to achieve some “early wins” in order to demonstrate the benefits of networks and secure broader engagement and ownership’. The current review identified the same. The second report by Cancer Australia [48] similarly identified the need for *clear and structured management arrangements* with one person acting as the overall lead coupled with inclusive multidisciplinary representation. Emphasis was also placed on *patient involvement*, comparable with the need for inclusive network membership involving patients and other stakeholders as well as a broad range of different healthcare professions, to ensure alignment of network priorities with the wider context, and the necessity for *formalised reporting requirements* to evaluate network quality improvement initiatives. This report further stressed the role of clinical networks in the dissemination of *evidence based practice* and promotion of *continuing professional development*, similar to the category of organisational learning and knowledge in the current review.

Consistent with the conclusions of other authors [49], results of the current review suggest those planning the establishment of clinical networks should take a combined ‘bottom-up’ and ‘top-down’ approach which maintains wide ranging multidisciplinary engagement and allows for decentralised decision-making to foster a collaborative ethos, coupled with effective and credible management and adequate administrative, human and technological resources to facilitate communication and collect relevant data.

### Strengths and limitations of the review

This is the first systematic review that has explicitly focused on the effectiveness of clinical networks to improve quality of care and patient outcomes. Like all systematic reviews, the conclusions of this review are limited by its scope and the range and quality of the research we have been able to uncover. Clinical networks are a relatively new phenomenon and it is difficult to identify relevant papers in any emerging field. This is especially true of research relating to clinical networks, which is often classified by clinical specialty. There is a lack of consistent terminology used to describe clinical networks, which was particularly evident in the earlier studies. To facilitate accurate identification of eligible studies, the researchers worked closely with a librarian to develop an iterative inclusive search strategy.

Clinical networks have many forms, are hard to define and operate in different contexts. Further, the reasons for setting up networks vary, as do their goals and activities. This is reflected in the diverse aims of the studies included in this review, which made it challenging to draw together the findings and any lessons to be learnt. We have strengthened the utility of this review by supplementing the relatively few quantitative empirical papers with qualitative research so as to be able to draw conclusions about the features necessary to enable clinical networks to be effectively used as implementation vehicles. To the best of our knowledge, this is the first time quantitative and qualitative results have been synthesised to evaluate clinical networks as an innovative way to organise healthcare delivery and what makes them successful.

### Future research questions and methods

This review highlights the gaps in the literature relating to the effectiveness of clinical networks in improving quality of care and patient outcomes, particularly a lack of empirical studies with rigorous study designs. The absence of randomised controlled trials and the few observational studies limits the ability to draw robust conclusions about whether clinical networks are more effective at improving health service delivery and patient outcomes than other approaches.

While results so far have been mostly positive, more studies are necessary to determine whether improvements in service delivery are translating into improved patient outcomes. Of note, only two studies were identified that explicitly measured change in patient outcome indicators. There is a need to strengthen the existing body of knowledge through higher level evidence from rigorously designed randomised controlled trials to test the impact of clinical network-led initiatives on both quality of care and patient outcome indicators. Where it is not possible to conduct internally and externally valid experimental studies within a real-world setting, observational studies with stronger methodological designs, like controlled before-and-after or interrupted time series studies, would improve upon the learning from the descriptive studies that are currently most prevalent in this area. Empirical studies are also needed to quantify what makes a network more or less successful and determine the features necessary to strengthen existing and effectively implement new clinical networks. While the qualitative articles provided significant narrative on what was perceived to make a network effective, this was rarely quantified or examined in any depth in the quantitative studies, highlighting a need for more mixed methods research. Furthermore, data on whether clinical networks are cost-effective vehicles to bring about change in a complex system is entirely lacking. Only one study reported on the economic impact of the implementation of a clinical network [4] and found no difference in the average cost per patient. More comprehensive economic analyses are required to evaluate whether clinical networks are a cost-effective way to improve quality and outcomes through coordinated integration of services and better flow of knowledge about best practice.

## Conclusions

There is some evidence that clinical networks can be vehicles to implement quality improvement initiatives. Given that clinical networks are being widely established, particularly in the UK and Australia, it is important to develop rigorous evidence to underpin future developments. Unfortunately, the generally low quality of quantitative effectiveness studies limits the ability to draw conclusions as to whether clinical networks can effectively improve the provision of healthcare and patient outcomes and whether these improvements can be maintained. Put simply, the research needs to ‘catch up’ with the operational developments in clinical networks. Our findings can, however, provide policymakers with some insight into the planning and implementation of a clinical network, specifically in regards to organisational structure, resourcing and interpersonal relationships, in order to increase the likelihood of success. Policymakers, clinicians and researchers need to work together in the implementation of clinical networks and their initiatives to design rigorous evaluations from the outset so as to be able to demonstrate their impact.

## Abbreviations

ACI, NSW Agency for Clinical Innovation; AUD, Australian Dollar; EPOC, Cochrane Effective Practice and Organisation Care Group; HPN, home parenteral nutrition; MI, myocardial infarction; NHMRC, National Health and Medical Research Council; NHS, National Health Service; NSW, New South Wales; PRISMA, Preferred Reporting Items for Systematic Reviews and Meta-Analyses; UK, United Kingdom; URR, urea reduction ratio; US, United States of America; USD, United States Dollar

## Additional files

Additional file 1: Title of data. Detailed description of systematic review methodology: Description of data: This attachment provides a detailed description of the methodology used for this systematic review including the identification of articles, quality and risk assessment, and data extraction and synthesis. (PDF 281 kb) Additional file 2: Title. Detailed findings of articles included in the systematic review: Description of data: This table provides details on the articles included in this systematic review, including their methods and findings. (PDF 367 kb) Additional file 3: Title. PRISMA Checklist: Description of data: This file provides details of where the required items in the PRISMA Checklist for systematic reviews are reported in the manuscript. (PDF 403 kb)

## References

[1] Braithwaite J, Goulston K (2004). Turning the health system 90° down under. Lancet.

[2] Goodwin N, Perri 6, Peck E, Freeman T, Posaner R (2004). Managing Across Diverse Networks of Care: Lessons from Other Sectors.

[3] Gale C, Santhakumaran S, Nagarajan S, Statnikov Y, Modi N, on behalf of the Neonatal Data Analysis Unit and the Medicines for Neonates Investigator G (2012). Impact of managed clinical networks on NHS specialist neonatal services in England: population based study. BMJ.

[4] Hamilton K, Sullivan F, Donnan P, Taylor R, Ikenwilo D, Scott A, Baker C, Wyke S (2005). A managed clinical network for cardiac services: set-up, operation and impact on patient care. International Journal of Integrated Care.

[5] Tolson D, McIntosh J, Loftus L, Cormie P (2007). Developing a managed clinical network in palliative care: a realistic evaluation. Int J Nurs Stud.

[6] Ray-Coquard I, Philip T, de Laroche G, Froger X, Suchaud J-P, Voloch A, Mathieu-Daude H, Fervers B, Farsi F, Browman G (2002). A controlled ‘before-after’ study: impact of a clinical guidelines programme and regional cancer network organization on medical practice. Br J Cancer.

[7] Ray-Coquard I, Philip T, de Laroche G, Froger X, Suchaud J-P, Voloch A, Mathieu-Daudé H, Lurkin A, Farsi F, Bertrand P (2005). Persistence of medical change at implementation of clinical guidelines on medical practice: a controlled study in a cancer network. J Clin Oncol.

[8] Haines M, Brown B, Craig J, D’Este C, Elliott E, Klineberg E, McInnes E, Middleton S, Paul C, Redman S (2012). Determinants of successful clinical networks: the conceptual framework and study protocol. Implement Sci.

[9] Stewart GJ, Dwyer JM, Goulston KJ (2006). The Greater Metropolitan Clinical Taskforce: an Australian model for clinician governance. Med J Australia.

[10] Sax Institute. What have the clinical networks acheived and who has been involved? 2006–2008: Retrospective study of the quality improvement activities of and participation in a taskforce of clinical networks. Sydney: Sax Institute; 2011.

[11] Alberta Health Services: Strategic Clinical Networks: A primer & working document (August 7, 2012 - V5). Alberta Health Services; 2012.

[12] Laliberte L, Fennell ML, Papandonatos G (2005). The relationship of membership in research networks to compliance with treatment guidelines for early-stage breast cancer. Med Care.

[13] Touati N, le Roberge D, Denis J, Cazale L, Pineault R, Tremblay D (2006). Clinical leaders at the forefront of change in health-care systems: advantages and issues. Lessons learned from the evaluation of the implementation of an integrated oncological services network. Health Serv Manag Res.

[14] Turrini A, Cristofoli D, Frosini F, Nasi G (2010). Networking literature about determinants of network effectiveness. Public Adm.

[15] NHS Commissioning Board (2012). The Way Forward: Strategic clinical networks.

[16] NHS Commissioning Board. Strategic Clinical Networks: Single Operating Framework. UK: NHS; 2012.

[17] Transcripts The Office of the Prime Minister of Australia PM. Prime Minister Minister for Health Lead Clinicians Groups to deliver a greater say for local health professionals. Prime Minister - Rudd, Kevin. Canberra: Australian Government, Department of the Prime Minister and Cabinet; 2010.

[18] National Institute of Clinical Studies (2006). Networks to support evidence implementation.

[19] Li LC, Grimshaw JM, Nielsen C, Judd M, Coyte PC, Graham ID (2009). Use of communities of practice in business and health care sectors: a systematic review. Implement Sci.

[20] Black A, Car J, Pagliari C, Anandan C, Cresswell K, Bokun T, McKinstry B, Proctor R, Majeed A, Sheikh A (2011). The impact of eHealth on the quality and safety of health care: a systematic overview. PLoS Med.

[21] Ekeland A, Dowes A, Flottorp S (2011). Effectiveness of telemedicine: a systematic review of reviews. International Journal of Informatics.

[22] Wade VA, Karnon J, Elshaug AG, Hiller JE (2010). A systematic review of economic analyses of telehealth services using real time video communication. BMC Health Serv Res.

[23] Cunningham F, Ranmuthugala G, Plumb J, Georgiou A, Westbrook J, Braithwaite J (2012). Health professional networks as a vector for improving healthcare quality and safety: a systematic review. BMJ Qual Saf.

[24] Moher D, Liberati A, Tetzlaff J, Altman DG, Group TP (2009). Preferred reporting items for systematic reviews and meta-analyses: the PRISMA statement. J Clin Epidemiol.

[25] Harden A, Brunton G, Fletcher A, Oakley A (2009). Teenage pregnancy and social disadvantage: systematic review integrating controlled trials and qualitative studies. BMJ.

[26] Thomas J, Harden A, Oakley A, Oliver S, Sutcliffe K, Rees R, Brunton G, Kavanagh J (2004). Integrating qualitative research with trials in systematic reviews. British Journal of Medicine.

[27] Liberati A, Altman D, Tetzlaff J, Mulrow C, Gotzsche P, Ioannidis J, Clarke M, Devereaux P, Kleijnen J, Moher D (2009). The PRISMA statement for reporting systematic reviews and meta-analyses of studies that evaluate health care interventions: explanation and elaboration. PLoS Med.

[28] EPPI-Centre. EPPI-Centre methods for conducting systematic reviews. In: Social Science Research Unit, UCL Institute of Education. London: University of London; 2007.

[29] Pope C, Ziebland S, Mays N (2000). Qualitative research in health care. Analysing qualitative data. BMJ.

[30] McCullough A, Scotland T, Dundas S, Boddie D (2014). The impact of a managed clinical network on referral patterns of sarcoma patients in Grampian. Scott Med J.

[31] Greene A, Pagliari C, Cunningham S, Donnan P, Evans J, Emslie-Smith A, Morris A, Guthrie B (2009). Do managed clinical networks improve quality of diabetes care? Evidence from a retrospective mixed methods evaluation. Qual Saf Health Care.

[32] Spence K, Henderson-Smart D (2010). Closing the evidence-practice gap for newborn pain using clinical networks. J Paediatr Child Health.

[33] McClellan WM, Frankenfield DL, Frederick PR, Flanders WD, Alfaro-Correa A, Rocco M, Helgerson SD (1999). Can dialysis therapy be improved? A report from the ESRD Core Indicators Project. Am J Kidney Dis.

[34] Tideman P, Tirimacco R, Senior D, Setchell J, Huynh L, Tavella R, Aylward P, Chew D. Impact of a regionalised clinical cardiac support network on mortality among rural patients with myocardial infarction. MJA. 2014;200(3):157–60. 10.5694/mja13.10645 24528431

[35] Ahgren B, Axelsson R (2007). Determinants of integrated health care development: chains of care in Sweden. International Journal of Health Planning & Management.

[36] Baker A, Wright M (2006). Using appreciative inquiry to initiate a managed clinical network for children’s liver disease in the UK. International Journal of Health Care Quality Assurance Incorporating Leadership in Health Services.

[37] Cunningham F, Ranmuthugula G, Westbrook J, Braithwaite J (2012). Net benefits: assessing the effectiveness of clinical networks in Australia through qualitative methods. Implement Sci.

[38] Hogard E, Ellis R (2010). An evaluation of a managed clinical network for personality disorder: Breaking new ground or top dressing?. J Eval Clin Pract.

[39] McInnes E, Middleton S, Gardner G, Haines M, Haerstch M, Paul C, Castaldi P (2012). A qualitative study of stakeholder views of the preconditions for and outcomes of successful networks. BMC Health Serv Res.

[40] Fleury M, Mercier C, Denis J-L (2002). Regional planning implementation and its impact on integration of a mental health care network. International Journal of Health Planning & Management.

[41] Addicott R (2008). Models of governance and the changing role of the board in the “modernised” UK health sector. Journal of Health Organization & Management.

[42] Addicott R, Ferlie E (2007). Understanding power relationships in health care networks. Journal of Health Organization & Management.

[43] Addicott R, McGivern G, Ferlie E (2007). The distortion of a managerial technique? The case of clinical networks in UK health care. Br J Manag.

[44] Addicott R, McGivern G, Ferlie E (2006). Networks, organizational learning and knowledge management: NHS cancer networks. Public Money & Management.

[45] Burnett S, Williams D, Webster L (2005). Knowledge support for interdisciplinary models of healthcare delivery: a study of knowledge needs and roles in managed clinical networks. Health Informatics Journal.

[46] Hallum N, Baxter J, O-Reilly D, McKee R (2010). Home parenteral nutrition in Scotland: frequecy of monitoring, adequacy of review and consequence for complication rates. Nutrition.

[47] Guthrie B, Davies H, Greig G, Rushmer R, Walter I, Duguid A, Coyle J, Sutton M, Williams B, Farrar S (2010). Delivering health care through managed clinical networks (MCNs): lessons from the North. Report for the National Institute for Health Research Service Delivery and Organisation programme.

[48] National Support and Evaluation Service - Siggins Miller. Managed Clinical Networks - a literature review. CanNET Cancer Service Networks National Demonstration Program. Canberra: Cancer Australia; 2008.

[49] McInnes E, Haines M, Dominello A, Kalucy D, Jammali-Blasi A, Middleton S, Klineberg E (2015). What are the reasons for clinical network success? A qualitative study. BMC Health Serv Res.

