# Supplementary material for: The effectiveness of clinical networks in improving quality of care and patient outcomes: a systematic review of quantitative and qualitative studies
Source: BMC Health Serv Res. 2016 Aug 8;16(1):360. doi: 10.1186/s12913-016-1615-z (PMC5018194; doi:10.1186/s12913-016-1615-z)
Supplement: Additional file 1: — Title of data. Detailed description of systematic review methodology: Description of data: This attachment provides a detailed description of the methodology used for this systematic review including the identification of articles, quality and risk assessment, and data extraction and synthesis. (PDF 281 kb) [file 12913_2016_1615_MOESM1_ESM.pdf]

Supplementary File 1 – Detailed description of systematic review methodology

Overall Approach

This systematic review was conducted in accordance with the PRISMA approach to ensure the transparent and complete reporting of our sensitive searching, systematic screening and independent quality assessment [1]. The concepts and overarching methods for systematic reviews [2] have been adapted to be applicable for a mixed methods systematic review [3, 4].

Eligibility – inclusion and exclusion criteria

Articles were eligible for inclusion in this review if:

i) The primary focus of the paper was on clinical networks in any healthcare setting (e.g. acute, primary, community, vertical integration)

ii) The networks corresponded with the category of network that would be included - that is a managed or non-managed clinical network

iii) The paper reported an outcome related to improvement of quality of care or patient outcomes (based on objective measures)

Excluded were:

i) Abstracts and titles with the term 'clinical network' that were not referring to actual clinical networks (e.g. clinical network guidelines, simulation studies for proposed networks, protocol papers detailing study plans of networks, information technology or infrastructure networks)

ii) Research networks

iii) Clinical trial networks

iv) Clinical guideline networks

v) Integrated service delivery networks (sometimes called regional networks or networked hospitals, Health Management Organisations and managed care organisations in the United States)

vi) Articles that used clinical networks as vehicles for samples for studies

vii) Articles that were not published in peer review journals (e.g. conference proceedings)

Identification and selection of publications

Initial search (1996-2010)

Authors BB and MH conducted the initial literature search with the assistance of a librarian/information scientist. Figure 1 (in the main text of the article) outlines the search process. We searched Medline, Embase and CINAHL to locate all research publications for the period 1996 to 2010 that focused on clinical networks. None of these databases have subject terms (i.e. MESH terms for Medline) that cover the concept of clinical networks so the search terms were developed based on 58 papers that were obtained through an initial search using the term 'clinical networks' and iterative searching. Box 1 contains the search terms used, restricted to the English language, with a year of publication between 1996 and 2010.

After duplicates were removed (N=57), researchers screened abstract titles (N=843) for inclusion. Abstracts with titles that had: a) the terms 'clinical network/s'; clinical specialty network (e.g. cancer network); or the word 'network'; and b) were referring to a clinical network, were included (N=151). In the case where a judgement could not be made on the basis of the abstract then the authors reviewed the whole publication to make a judgement on whether it should be included in the review.

Box 1 – Search terms used to identify articles for this systematic review

EMBASE

Query 1 *National Health Service/ or *public relations/ or *Integrated Health Care System/ or *managed care/ or exp *cooperation/ or exp *patient care/ or exp *health care quality/ or exp *disease management/ or *health care management/ or exp Health Care System/

Query 2 ((regional adj2 **network***) or (national adj2 **network***) or clinical **network***).mp. [mp=ti, ab, sh, hw, tn, ot, dm, mf, nm

Query 3 Combine queries 1 and 2

Query 4 Limit 3 to (english language and yr="1996 - 2008")

MEDLINE

Query 1 *state medicine/ or *interinstitutional relations/ or *delivery of health care integrated/ or *managed care programs/ or *cooperative behavior/ or exp patient care management/ or exp "Quality of Health Care"/

Query 2 ((regional adj2 network*) or (national adj2 network*) or clinical network*).mp.

Query 3 Combine queries 1 and 2

Query 4 Limit 3 to (english language and yr="1996 - 2008")

CINAHL

Query 1 mm National Health Programs or mm Interinstitutional Relations or mm Health Care Delivery, Integrated or mm Managed Care Programs or mm Cooperative Behavior or mm Patient Care+ or mm Quality of Health Care+ or mm Disease Management or mm Health Care Delivery+

Query 2 ((regional adj2 network*) or (national adj2 network*) or clinical network*).mp. [mp=ti, ab, sh, hw, tn, ot, dm, mf, nm

Query 3 Limit 2 to (english language and yr="1996 - 2008")

Two authors (MH, BB) independently reviewed the identified abstracts for eligibility and cross-checked their classifications. There was 96% agreement between the authors' initial

codes (145/151) and after discussion there was 100% agreement on whether the abstract should be included (n=89).

After excluding abstracts for which the full text was unavailable (n=28) and including publications identified through screening of reference lists of included articles (n=3), two authors (MH, BB) independently reviewed these full text articles (n=64) and cross-checked their classifications to confirm whether the publication should be included in the analysis based on the criterion of whether the study was focused on a mandatory or non-mandatory clinical network. There was 94% agreement between reviewers (60/64) and, following discussion, 23 articles were excluded. The remaining 41 eligible papers were coded into empirical (n=20) and commentary contributions (n=21). Empirical studies were defined as original research and presented new data - either qualitative or quantitative. The commentary pieces were excluded. As a further quality assurance measure, a third author (CP) assessed the eligibility of the 20 empirical studies against the above criteria. This resulted in three further exclusions with reasons.

The remaining 17 empirical studies were included regardless of country, number of networks studied, clinical focus of the networks, study design or outcomes assessed in relation to the networks.

Updated search (2011-2014)

Following the steps outlined above, two authors (BB, CP) performed an updated literature search for the period covering 1 January 2011 to 30 September 2014 (PubMed and CINAHL were searched to update the search from 1 January 2013 to 30 September 2014). A separate search using the search term "clinical network" was also performed given the more frequent

use of this term in recent years. The search procedure is outlined in Figure 2. Following the same procedure as the initial search, 2,035 titles were screened, duplicates removed and assessed for eligibility, with 95 abstracts remaining. Based on the inclusion and exclusion criteria above and excluding commentary articles, we excluded 44 abstracts, leaving 51 eligible abstracts. Both authors independently reviewed 50 full-text publications (one full-text was unavailable) to determine whether they should be included in the review. Forty-three articles were excluded, as they did not meet the eligibility criteria. Queries were resolved by consultation with a third author (MH). After discussion, there was 100% agreement between the three authors on which articles met the eligibility criteria for inclusion. Reference lists of the included papers and relevant commentary papers were reviewed for inclusion of additional eligible articles, but none meeting our criteria were found. The updated search yielded an additional five papers to be included in this review.

With 17 articles from the initial search and 5 from the updated search, a total of 13 qualitative and 9 quantitative studies were included over our search period from 1996 to 30 September 2014.

Quality and assessment of risk bias

The risk of bias and quality assessment of the quantitative studies and qualitative studies were assessed separately [2, 5].

Quantitative Studies

The quantitative study designs were assessed on the basis of whether they would meet the study design acceptable for a Cochrane Effective Practice and Organisation of Care Group (EPOC) review with those being: a) patient or cluster randomised control trials; b) non-

randomised cluster control trials; c) controlled before and after studies; and d) interrupted time series [6, 7]. Given the lack of high quality study designs found in the included articles, study designs were coded into the followed grades of evidence used previously for a communities of practice review [8]:

1. Experimental
2. Quasi-experimental studies (controlled trials, time series, controlled before and after designs)
3. Observational designs (before and after studies, cross-sectional studies).

The assessment of the quality of the methods and reporting drew on elements of EPOC and the Agency for Healthcare Research and Quality [6, 9]:

- Was the study free from selective outcome reporting? (yes/no/unclear)
- For comparative studies, was the control/comparison group used equivalent to the intervention group? (yes/no) (where appropriate)
- For non-comparative studies, were the cases representative (i.e. all eligible cases over a defined period of time, all cases in a defined catchment area, all cases in a defined hospital, clinic or group, or an appropriate sample of those cases)? [10] (yes/no) (where appropriate)
- Was there a clear description of the exposure or intervention? (yes/no)
- Was the study adequately protected against contamination? (yes/no/unclear) (where appropriate)
- Statistical analysis – were the methods appropriate and was reporting adequate? (yes/no)
- Was there a declaration of funding or sponsorship? (yes/no)
- Was the study free from other risks of bias? (yes/no)

The studies were grouped into three categories on the basis of quality of methods and reporting [11]:

- High quality – design and conduct of study address risk of bias, appropriate measurement of outcomes, appropriate statistical and analytical methods, low drop-out rates, adequate reporting;
- Moderate quality – do not meet all criteria for a rating of good quality but no flaw is likely to cause major bias, some missing information;
- Low quality – significant biases including inappropriate design, conduct, analysis or reporting, large amounts of missing information, discrepancies in reporting.

Two authors (BB, CP) independently assessed each quantitative study against the criteria above. There was 50% agreement (5/10 articles) and through discussion there was 90% agreement (9/10 articles) with final ratings given to 8 articles (see Table 1). A third author (MH) resolved one instance where there was disagreement and two instances where additional input was sought. The authors agreed that observational articles would not be given a "high" quality rating even when bias was minimised in the study due to the inherent flaws of an observational study design. At this stage, one article in question was deemed to be ineligible and excluded from this review. There was 100% agreement on the quality assessment rating of the nine included articles between the three authors.

<u>Qualitative Studies</u>

There is lack of consensus about how to assess risk of bias for qualitative studies [12]. For this review we considered that assessing the validity of the methods and quality of the reporting was the most appropriate approach to take [13, 14]. To do this, we used nine criteria

to assess the quality of qualitative studies recently developed by Harden and colleagues [4] and two criteria on the extent to which the 'participant voice' [15] was elucidated using a definition suggested by Mays and Pope [13] (see Box 2).

Box 2 - Criteria used to assess the quality of the qualitative studies.

Quality of reporting [4]

1. Were the aims and objectives clearly reported?
2. Was there an adequate description of the context in which the research was carried out?
3. Was there an adequate description of the network and the methods by which the sample was identified and recruited?
4. Was there an adequate description of the methods used to collect data?
5. Was there an adequate description of the methods used to analyse data?

Use of strategies to increase reliability and validity [4]

6. Were there attempts to establish the reliability of the data collection tools (for example, by use of interview topic guides)?
7. Were there attempts to establish the validity of the data collection tools (for example, with pilot interviews)?
8. Were there attempts to establish the reliability of the data analysis methods (for example, by use of independent coders)?
9. Were there attempts to establish the validity of data analysis methods (for example, by searching for negative cases)?

Quality of the application of the methods [13]

10. The extent to which qualitative studies are grounded in and reflect study participants' perspective and experiences (as evidenced by the use of supporting quotes)
11. Whether the studies produce also rich or 'thick' descriptions of the investigation and explanatory insights rather than 'thin' descriptions or flat summaries of the findings.

We grouped these studies into three categories on the basis of quality in accordance with the approach used by Harden and colleagues [4] and the Cochrane qualitative research methods group [16]. Arbitrary cut offs were selected as:

- High quality – those meeting 8 or more criteria
- Medium quality – those meeting between 5 and 7 criteria
- Low quality – those meeting fewer than five criteria

Data extraction and synthesis

Given the lack of high quality evidence from randomised controlled trial data, we adopted a pragmatic approach of examining all available evidence from primary observational studies, and assessing study quality within this lower level of the evidence hierarchy. Studies were first categorised as either qualitative or quantitative. Quantitative papers were then further categorised according to the focus of the study linked to the review objectives into two categories:

1. Improving quality of care: These papers examined whether clinical networks were successful in improving the delivery of health care.
2. Improving patient outcomes: These papers examined whether reorganisation into clinical networks or interventions implemented by networks were effective in improving patient outcomes.

Qualitative methods were used to thematically analyse and synthesise textual data extracted from the qualitative studies [17]. Two authors (BB and CP) independently identified the focus of the qualitative papers and categorised them into four themes. As several papers could have been classified under more than one theme, articles were categorised on the basis of the most prominent theme. The four themes were:

1. Features and outcomes of effective networks: These papers examined what features of a network enabled it to be successful, and what successful networks have achieved.
2. Network implementation: These articles described the process of implementing a clinical network and the key lessons learned from the implementation process.
3. Organisational structure: These articles looked at how networks were structured and how its structure impacted the way the network worked (namely, the network's ability to achieve its desired outcomes).
4. Organisational learning and knowledge: These articles examined the organisational learning and education role of clinical networks.

Due to the heterogeneity of the included studies, data were extracted directly into a data extraction table. Information was extracted on: i) country; ii) description of network studied; iii) description of the sample and size in terms of networks and participants; iv) study aim; v) intervention (quantitative studies); vi) design; vii) data collection method; viii) outcomes assessed; ix) results. One author (BB) extracted all the information from the initial search on the basis of what was available in the publications and a second (CP) checked all the extracted information. There was majority agreement between the reviewers on the data extracted and queries were resolved through consensus. For the updated search, two authors (BB, CP) extracted information from the articles and agreed on the data extracted through consensus. The main findings of the quantitative and qualitative studies were first examined separately, and then integrated to identify recurrent themes and findings to enable conclusions to be drawn.

Due to the heterogeneity of the included quantitative studies and their outcomes, results were reported narratively. Key outcomes demonstrating the effectiveness of clinical networks were

reported. Qualitative methods were used to synthesise textual data extracted from the qualitative studies. Results from the quantitative narrative analysis were then integrated with the qualitative synthesis in the discussion to identify recurrent themes and findings to enable conclusions to be drawn. Details on the findings of each of the included articles can be found in Additional File 2.

References

1. Moher D, Liberati A, Tetzlaff J, Altman DG, Group TP: **Preferred Reporting Items for Systematic Reviews and Meta-Analyses: The PRISMA Statement**. *Journal of Clinical Epidemiology* 2009, **62**:1006-1012.
2. Liberati A, Altman DG, Tetzlaff J, Mulrow C, Gøtzsche PC, Ioannidis JPA, Clarke M, Devereaux PJ, Kleijnen J, Moher D: **The PRISMA statement for reporting systematic reviews and meta-analyses of studies that evaluate health care interventions explanation and elaboration**. *Journal of Clinical Epidemiology* 2009, **62** e1-e34.
3. Thomas J, Harden A, Oakley A, Oliver S, Sutcliffe K, Rees R, Brunton G, Kavanagh J: **Integrating qualitative research with trials in systematic reviews**. *BMJ* 2004, **328**:1010-1012.
4. Harden A, Brunton G, Fletcher A, Oakley A: **Teenage pregnancy and social disadvantage: systematic review integrating controlled trials and qualitative studies**. *BMJ* 2009, **339**:1-11.
5. Evidence for Policy and Practice Information and Coordinating Centre: **EPPI-Centre Methods for Conducting Systematic Reviews**. In. Edited by EPPI-Centre: Social Science Research Unit, Institute of Education, University of London; 2007.
6. **EPOC Resources for review authors** [http://epoc.cochrane.org/epoc-specific-resources-review-authors]
7. Eccles M, Grimshaw J, Campbell M, Ramsay C: **Research designs for studies evaluating the effectiveness of change and improvement strategies**. *Quality & Safety in Health Care* 2003, **12**(1):47-53.
8. Li LC, Grimshaw JM, Nielsen C, Judd M, Coyte PC, Graham ID: **Use of communities of practice in business and health care sectors: A systematic review**. *Implement Sci* 2009, **4**(27):1-9.
9. West S, King V, Carey TS, Lohr KN, McKoy N, Sutton SF, Lux L: **Systems to rate the strength of scientific evidence**. In. Rockville, MD: Research Triangle Institute - University of North Carolina Evidence-based Practice Centre; 2002.
10. Wells GA, Shea B, O'Connell D, Peterson J, Welch V, Losos M, Tugwell P: **The Newcastle-Ottawa Scale (NOS) for assessing the quality of nonrandomised studies in meta-analysis**. In.
11. Helfand M, Balshem H: **Principles in developing and applying guidance**. In: *Methods Reference Guide for Comparative Effectiveness Reviews [posted August 2009]*. Edited by Quality AfHRa. Rockville, MD: Agency for Healthcare Research and Quality; 2009.

12. Cochrane Qualitative Research Methods Group: **Chapter 6 - Critical appraisal of qualitative research**. In.: Cochrane Qualitative Research Methods Group; 2009.
13. Mays N, Pope C: **Assessing quality in qualitative research**. *BMJ* 2000, **320**:50-52.
14. Mays N, Pope C, Popay J: **Systematically reviewing qualitative and quantitative evidence to inform management and policy-making in health care field**. *Journal of Health Services Research & Policy* 2005, **10**(1):6-20.
15. Popay J, Rogers A, Williams G: **Rationale and Standards for the Systematic Review of Qualitative Literature in Health Services Research**. *Qualitative Health Research* 1998, **8**(3):341-351.
16. Hannes K: **Chapter 4: Critical appraisal of qualitative research**. In: *Supplementary Guidance for Inclusion of Qualitative Research in Cochrane Systemicatic Reviews of Interventions.* Edited by Noyes J, Booth A, Hannes K, Harden A, Harris J, Lewin S, Lockwood C, 1 edn: Cochrane Collaborative Qualitative Methods Group; 2011.
17. Pope C, Ziebland S, Mays N: **Qualitative research in health care. Analysing qualitative data.** *BMJ* 2000, **Jan 8**(320(7227)):114-116.
