# Supplementary material for: The effectiveness of clinical networks in improving quality of care and patient outcomes: a systematic review of quantitative and qualitative studies
Source: BMC Health Serv Res. 2016 Aug 8;16(1):360. doi: 10.1186/s12913-016-1615-z (PMC5018194; doi:10.1186/s12913-016-1615-z)
Supplement: Additional file 2: — Title. Detailed findings of articles included in the systematic review: Description of data: This table provides details on the articles included in this systematic review, including their methods and findings. (PDF 367 kb) [file 12913_2016_1615_MOESM2_ESM.pdf]

Supplementary File 2 – Detailed findings of articles included in the systematic review

Quantitative Articles

Reference	Country	Type of Network	Sample	Study Aim, Design, Method and Indicators	Primary Results
▪ **Improving Quality of Care**					
Gale et al 2012	UK	Managed clinical network for neonatal services	*Before reorganisation:* from report of the Confidential Enquiry into Stillbirths and Death in Infancy (CESDI) Project 27/28, data from 1 Sep 1998 to 30 Aug 2000. Data was from England, Wales and Northern Ireland and was not disaggregated. *After reorganisation:* from National Neonatal Research Database held by the Neonatal Data Analysis Unit, data from 1 Jan 2009 to 31 Dec 2010.	**Aim**<br>To assess the impact of reorganisation of neonatal specialist care services in England following the formation of managed clinical networks, specifically the impact on access to specialist care for pre-term births<br>**Intervention**<br>National reorganisation of neonatal services in England into managed clinical neonatal networks<br>**Design**<br>Population-wide observational comparison of outcomes before and after the establishment of managed clinical neonatal networks.<br>**Method**<br>• Analysis of data on live births born at 27-28 weeks' gestation held by the Neonatal Data Analysis Unit and CESDI Project 27/28<br>**Indicators**<br>• Proportion of babies born at hospitals providing the highest volume of neonatal	▪ The proportion of babies delivered at 27-28 weeks' gestation in hospitals with the highest specialist care activity increased significantly from 18% (England, Wales and Northern Ireland) to 49% (England only) (risk difference 31%, 95% CI: 28 to 33; odds ratio 4.30, 3.83 to 4.82; P<0.001), indicating success of the networks in increasing high risk transfers<br>▪ The proportion of babies undergoing acute and late postnatal transfer in England increased significantly from 7% to 12% and 18% to 22%, respectively ($\chi 2$ P<0.001)<br>▪ No difference in proportion of transferred twins/triplets (33% vs 29%, odds ratio 0.86, 95% CI: 0.50 to 1.46; P=0.57)<br>▪ Survival in England increased from 88% to 94% (risk difference 5.6% (95% CI: 4.2 to 7.0); odds ratio 2.00 (95% CI: 1.67 to 2.40); P<0.001)<br>▪ However given over half of the study population were not delivered at a centre providing the highest volume of neonatal intensive care activity, poor adherence to the guidelines of the National Audit Office and National Institute for Health and Clinical Excellence is ongoing, underlining the limitations of a major reorganisation of one aspect of service provision rather than the entire pathway of care.

Reference	Country	Type of Network	Sample	Study Aim, Design, Method and Indicators	Primary Results
			This data was from England only.	specialist care<br>• Proportion of acute transfer and/or late transfer<br>• Proportion of babies in multiple births separated by transfer	
Greene et al 2009	UK	Tayside Diabetes Managed Clinical Network	13,527 patients with diabetes in the region treated by 72 general practices and 2 district hospitals.<br>36 in-depth interviews with a purposive sample of people with high and low commitment to managed clinical networks: Network core management group (n=9); GPs (n=3); Hospital professionals (n=8); patients (n=4); patient representatives and Trust managers (n=5)	**Aim**<br>To evaluate the form and impact of quality improvement (QI) strategies used by the Tayside Diabetes Managed Clinical Network between 1998 and 2005<br>**Intervention**<br>Progressive implementation of multiple quality improvement strategies including; guideline development and dissemination; education; clinical audit, feedback and benchmarking; encouragement of multidisciplinary team working; task redesign; and care pathway redesign<br>**Design**<br>▪ Retrospective observational mixed-methods evaluation<br>**Method**<br>▪ Analysis of network documents (annual reports, planning documents, minutes of network meetings), observation of meetings and qualitative semi-structured interviews with multidisciplinary team	▪ Simple process indicators such as measuring glycated haemoglobin, blood pressure and cholesterol rapidly improved, while there was slow continuous improvement for complex processes that required more intensive professional education or redesign of care pathways such as assessment of foot vascular and neurological status and retinal screening.<br>▪ Improvements were greater for type 2 than type 1 diabetes.<br>▪ Between 2002 and 2006, there was a 13% (95%CI: 11.6% to 14.1%; $p<0.001$) fall in the proportion of newly diagnosed patients with type 2 diabetes attending the hospital in the previous 15 months. However the number of patients treated in hospital remained unchanged due to rising prevalence.<br>▪ Network organisation and leadership with a clear vision for care were important facilitators in delivering QI in particular, achieving widespread clinical engagement through persuasion and appeal to shared professional values by clinical leaders.<br>▪ Information technology played a supportive role but was not perceived to deliver QI by itself.

Reference	Country	Type of Network	Sample	Study Aim, Design, Method and Indicators	Primary Results
				members and patients<br>▪ Analysis of impact of QI strategies using data extracted from the regional diabetes register at two time points – 1/1/1998 and 1/1/2005<br>**Indicators**<br>▪ 17 indicators of clinical processes and outcomes for patients with type 1 and type 2 diabetes (e.g. blood pressure measured, foot neurological status assessed, mean glycated haemoglobin %)<br>▪ Shifting care for uncomplicated type 2 diabetes into primary care, measured by rates of hospital referral for newly diagnosed patients	
Hamilton et al 2005	Scotland	Managed clinical network for cardiac services	N = 202 myocardial infarction patients < 76 years old admitted between 1st July 2000 and 30th June 2002 (97 prior to launch of the network) and 105 after launch of the network) in Dumfries and Galloway, South West	**Aim**<br>To investigate the setup and operation of a managed care network for cardiac services, and assess its impact on quality of patient care and resource implications<br>**Intervention**<br>Establishment of a managed clinical network for cardiac services in a predominantly rural area in South West Scotland<br>**Design**<br>Quasi-experimental study design (interrupted time series) - Single case study using process evaluation and observational	▪ The network brought clinicians, patients and managers together to redesign services.<br>▪ There was statistically significant improvement in 2 out of 16 clinical care indicators: immediate aspirin administration (Regression coefficient= -35.9; p=0.037) & pain to needle times (Regression coefficient= -1.207; p=0.051)<br>▪ There was non-significant improvement in 9 other indicators.<br>▪ Changes were not noticeable until after a 2 year start-up period<br>▪ No improvement in 5 indicators.<br>▪ Set-up costs of the MCN were £52,615 during its pilot year. A further £50,000 was allocated for administrative support and time of the clinical lead following the MCN’s launch. These costs are underestimates due to the difficulty in obtaining

Reference	Country	Type of Network	Sample	Study Aim, Design, Method and Indicators	Primary Results
			Scotland	before and after comparison<br>**Method**<br>▪ Document Reviews<br>▪ Interviews with two patients and a random sample of 12 health service personnel<br>▪ Analysis of routinely collected clinical data<br>**Indicators**<br>▪ Process evaluation of network setup – how was the network set up, how did it operate, what did it do? – clinical leadership, scepticism & lack of support, collaboration, communication, quality, equity<br>▪ Outcome evaluation of network impact – impact on 16 quality of patient care indices, including percentage of patients receiving: immediate aspirin, thrombolysis, discharge medication, cardiac rehabilitation, secondary prevention at 6 months post MI<br>▪ Economic evaluation of cost of setup and operation of network – what were the resource implications of the network?	data.<br>▪ No significant difference in hospital cost of care (£2,055 before and £2,053 after launch of MCN), length of stay or resource use.<br>▪ An energetic lead clinician and change in structure of the network from a flat internal structure to mainly hierarchical was crucial to the stability and acceptability of the network, leading to its successful implementation.
McCullough et al 2014	Scotland	Scottish Sarcoma Managed Clinical	158 patients identified	**Aim**<br>To determine whether the	▪ Prior to establishment of the network more patients were referred directly to the sarcoma

Reference	Country	Type of Network	Sample	Study Aim, Design, Method and Indicators	Primary Results
		Network (SSMCN)	through a database of all patients with histopathology reports presenting with sarcomas of the trunk or extremity in Grampian between 1991 and 2009 (79 before establishment of the network, 79 after; the network was established in 2004). An additional 144 records (48% of all records) were unavailable due to medical record destruction; most of these were from the before period.	establishment of the Scottish Sarcoma Network improved the quality of diagnosis, treatment and care of sarcoma patients<br>**Intervention**<br>Establishment of the Sarcoma Managed Clinical Network. Key interventions included facilitating national multidisciplinary discussion of all sarcoma cases, registering case details and provision of care by a multidisciplinary team.<br>**Design**<br>Retrospective observational comparison before and after the establishment of the sarcoma clinical network<br>**Method**<br>• Cohort analysis of patient records pre- and post-establishment of the network using administrative datasets and medical records<br>**Indicators**<br>▪ Referral to specialised sarcoma services<br>▪ Time to specialist review,<br>▪ Preoperative magnetic resonance imaging scanning<br>▪ Proportion of patients undergoing investigation with MRI scan prior to excision of sarcoma<br>▪ Proportion of patients undergoing appropriate	service by GPs, while subsequently greater numbers presented from other hospital specialists with referral numbers peaking in 2005 and 2006 following the initiation of the network.<br>▪ More patients were seen by more specialities after establishment of the network.<br>▪ Time interval from receipt of a referral to initial assessment by the service improved from a median of 19.5 days to 10 days after the SSN was established (p=0.016). However the interval between initial GP consultation and initial assessment by service increased from 35 to 41 days (p=0.57).<br>▪ Patients undergoing investigation with a magnetic resonance imaging (MRI) scan prior to excision of the sarcoma, increased from 67% to 86% after the establishment of the network (p = .0009)<br>▪ There was an increase in the number of patients undergoing appropriate biopsy from 57% to 79% (p=0.006).<br>▪ Data were available on the adequacy of surgical margins in 69 patients in each group. Resection margins were grouped into complete and incomplete margins. Prior to the network, 33 (48%) patients had documented complete resection and 36 (52%) were documented as incomplete. Post network this has increased to 56 (81%) complete margins and 13 (19%) (p <0.001).

Reference	Country	Type of Network	Sample	Study Aim, Design, Method and Indicators	Primary Results
				biopsy<br>▪ Complete margins achieved at surgical resection	
Ray-Coquard et al 2002	France	Regional cancer network of hospitals	**Experimental group – patients at 4 hospitals (private and public)**<br>**Control group – patients at 3 hospitals (private and public)**<br><br>**Breast Cancer**<br>Women with newly referred localised breast cancer.<br>Experimental Group:<br>1994 N = 282<br>1996 N = 346<br><br>Control Group:<br>1994 N = 194<br>1996 N = 172<br><br>**Colon Cancer**<br>All new patients with colon cancer.<br>Experimental Group -:<br>1994 N = 95	**Aim**<br>To assess the compliance of medical practice with clinical practice guidelines in hospitals in a region with a regional cancer network and a matched region without a network at two time points.<br>**Intervention**<br>Implementation of clinical practice guidelines (CPGs) through a regional clinical network<br>**Design**<br>Controlled before and after study with hospitals in a matched control region<br>**Method**<br>▪ Analysis of institutional medical records from patients pre- and post-implementation of clinical practice guidelines<br>**Indicators**<br>▪ The number of overall treatment sequences judged to conform with clinical practice guidelines or to be evidence-based<br>▪ For breast cancer procedures the overall treatment sequence included: initial examination; surgery; chemotherapy; radiotherapy;	▪ Compliance with guidelines for the overall treatment sequence was significantly higher in 1996 (36%; 95%CI: 30-42) than in 1994 (12%; 95%CI: 8-16) in the experimental group for breast cancer (p<0.001).<br>▪ Compliance with guidelines for the overall treatment sequence was significantly higher in 1996 (46%; 95%CI: 30-54) than in 1994 (14%; 95%CI: 7-21) in the experimental group for colon cancer (p<0.001).<br>▪ There was no change in the compliance rate in the control group for both cancers:<br>▪ The number of medical decisions that conformed to clinical practice guidelines or judged to be based on scientific evidence was significantly higher in the experimental groups after the intervention. There was no significant change in the control groups.<br>▪ Breast cancer: 62% (95%CI: 54-64) in 1996 vs 47% (95%CI: 41-53) in 1994 (p<0.001)<br>▪ Colon cancer: 86% (95%CI: 80-92) in 1996 vs 74% (95%CI: 65-82) in 1994 (p<0.001)

Reference	Country	Type of Network	Sample	Study Aim, Design, Method and Indicators	Primary Results
			1996 N = 94<br><br>Control Group:<br>1994 N = 89<br>1996 N = 118	hormonal therapy and follow-up<br>▪ For colon cancer procedures the overall treatment sequence included: initial examination; surgery; chemotherapy and follow-up	
Ray-Coquard et al 2005	France	Regional cancer network of hospitals	All new patients with colon cancer and breast cancer at two audit points.<br><br>**Experimental group – 4 hospitals (private and public)**<br>**Control group – 3 hospitals (private and public)**<br><br>**Colon Cancer**<br>Experimental group<br>1996 N = 177<br>1999 N = 200<br><br>Control group<br>1996 N = 118<br>1999 N = 100<br><br>**Breast cancer**<br>Experimental	**Aim**<br>To evaluate the persistence of conformity to clinical practice guideline (CPG) recommendations in a cancer network through an audit of medical practice records<br>**Intervention**<br>Implementation of CPG through a clinical network initiated in 1995<br>**Design**<br>Quasi-experimental study design - Controlled transversal study in experimental (cancer network) and control (no cancer network) groups<br>**Method**<br>▪ Analysis of institutional medical records at two audit points<br>**Indicators**<br>• The number of 825 assessable overall treatment sequences judged to conform with clinical practice guideline recommendations or to be evidence based<br>• The overall treatment	• Amongst breast cancer patients, compliance of medical decisions with CPG recommendations in the experimental group was similar for both periods (40%; 95%CI: 35-45 in 1996 vs 36%; 95%CI: 31-41 in 1999; p=0.25). Compliance was also the same in the control group (7% in 1996 vs 4% in 1999; p=0.99). Of note, the stratified analysis showed that only cancer centres maintained their initial compliance for surgical procedures (>85% and 75% in the experimental and control groups, respectively) whereas compliance rates decreased to less than 70% in all other institutions.<br>• For breast cancer patients, the proportion of medical decisions that were consistent with CPG or based on scientific evidence remained at the same level between 1996 (50%; 95%CI: 45-55) and 1999 (44%; 95%CI: 39-49) (p=0.01). In the control group, these results were 8% in 1996 (95%CI: 4-12) vs 10% (95%CI: 6-14) (p=0.58).<br>• Amongst colon cancer patients, compliance of medical decisions with CPG recommendations in the experimental group increased between 1996 (56%; 95%CI: 49-63) and 1999 (73%; 95%CI: 67-79) (p=0.003). Compliance was also the same in the control group (7% in 1996 vs 4% in 1999; p=0.99). Compliance was also higher in the control group (38%; 95%CI: 30-48 in 1996 vs

Reference	Country	Type of Network	Sample	Study Aim, Design, Method and Indicators	Primary Results
			group<br>1996 N = 444<br>1999 N = 381<br><br>Control group<br>1996 N = 172<br>1999 N = 204	sequence included decisions for each type of procedure individually (surgery, radiotherapy, chemotherapy, hormone therapy, initial examination, and follow-up)	67%; 95%CI: 58-76 in 1999; p<0.001). Stratified analyses showed that the compliance rate of the overall treatment sequence was higher in 1999 than in 1996 for any stage of disease in the experimental group, but only for the metastatic stage in the control group (and not for the localised group, p=0.11).<br>• For colon cancer patients, the proportion of medical decisions that were consistent with CPG or based on scientific evidence remained at the same level between 1996 (83%; 95%CI: 76-89) and 1999 (75%; 95%CI: 69-81) (p=0.49). In the control group, compliance increased from 59% in 1996 (95%CI: 50-67) to 68% (95%CI: 59-77) (p=0.01).<br>• The authors concluded that in this network, clinical practice guidelines were able to produce sustained improvements in adherence to medical practice over time compared with a control region.
Spence & Henderson-Smart 2011	Australia	Australian and New Zealand Neonatal Network	All neonatal nurses, midwives, neonatologists, junior medical staff, allied health and families providing care for newborn infants in 23 tertiary institutions with a neonatal intensive care	**Aim**<br>To establish a process incorporating a team approach for using evidence to support practice change and prove its effectiveness in closing the evidence practice gap for newborn pain<br>**Intervention**<br>The implementation model used a clinical network with state facilitators, local champions and project teams. Interventions included:<br>▪ Resource documents distributed to each	• Statistically significant increase in the percentage of attending staff aware of an available clinical practice guideline for management of newborn pain (61% to 86%; p=0.000)<br>• 21% improvement in the number of infants receiving sucrose for procedural pain (p<0.005).<br>• Use of pain assessment tool increased from 14% to 22%, although was still under-utilised.<br>• 56% (13/23) of units introduced the use of a pain assessment tool into practice.<br>• Distribution of information resulted in an increase in family awareness that their infant can experience pain and strategies to manage the pain (19% to 57%, p=0.000). The proportion of families that received any form of printed

Reference	Country	Type of Network	Sample	Study Aim, Design, Method and Indicators	Primary Results
			unit, one special care unit and special care nurseries in 9 district hospitals across 8 Australian states participated in the project. Neonates of all gestational ages and post-natal ages who were in-patients in each unit during the audits were included as part of a quality improvement project.	participating unit<br>▪ Educational workshops on critical appraisal<br>▪ Audit and feedback at baseline and after 18 months<br>▪ Point of care reminders<br>▪ Posters and parent information brochures<br>▪ Clinical practice guideline<br>**Design**<br>Observational before-and-after study.<br>**Methods**<br>▪ Surveys of clinical practices<br>▪ Prospective collection of data from participating units at baseline and 18 months after commencement of the project<br>▪ Audit of the use of a pain assessment tool for ventilated neonates 3 months prior to the project and 2 years after commencement<br>▪ Audits with families of infants<br>**Indicators**<br>▪ Use of sucrose or breastfeeding for procedural pain<br>▪ Use of pain assessment tool for ventilated neonates<br>▪ Parents awareness of their infant's pain	information doubled from 8% to 17%.<br>• Some targets were not met during the two year study period but a process for sustainability was established through the network to allow that to occur in the future
▪ **Improving patient outcomes**					
McClellan et al 1999	US	End Stage Renal Disease (ESRD)	Within each ESRD network,	**Aim**<br>To assess the association between	▪ At baseline there was substantial variation between networks in URR, with mean age,

Reference	Country	Type of Network	Sample	Study Aim, Design, Method and Indicators	Primary Results
		Networks	each year between 1994 and 1997, an annual random sample was selected of Medicare beneficiaries aged 18 and over receiving haemodialysis in the fourth quarter of 1993 – 1996. Network specific interventions were conducted with a 10% sample of treatment centres in each of the18 ESRD Networks	quality improvement interventions and change in haemodialysis adequacy using network specific interventions<br>**Intervention**<br>Network specific interventions included education on quality improvement, workshops, on-site assistance, distribution of an algorithm for assessing dialysis adequacy and distribution of clinical practice guidelines. National intervention reports were generated, comparing URRs by network, distribution of guidelines and patient education.<br>**Design**<br>Evaluation of a population-based, prospective quality improvement intervention.<br>**Method**<br>• Completion of a network-specific activities survey to ascertain interventions undertaken by each network, and an annual patient-level survey (completed by staff at each dialysis facility) to inform calculation of URRs.<br>• Analysis of haemodialysis adequacy before and after national and network-specific quality improvements interventions<br>**Indicators**	proportions of patients who were male or black, and distribution of causes of ESRD.<br>▪ Mean URR increased from 63% in 1993 to 67% in 1996 ($p<0.001$).<br>▪ The proportion of under-dialysed patients decreased from 56.6% in 1993 to 31.7% in 1996 ($p<0.0001$).<br>▪ Prolonged supervision in selected facilities was associated with an increased rate of improvement in URR from 62.1% at baseline to 67.7% after the intervention ($p<0.001$).

Reference	Country	Type of Network	Sample	Study Aim, Design, Method and Indicators	Primary Results
				• Network-specific Urea reduction ratios (URRs)	
Tideman et al 2014	Australia	Integrated cardiac support network (Integrated Cardiovascular Clinical Network – ICCNet)	29,623 independent contiguous episodes of MI identified through hospital administrative data and statewide death records from 1 July 2001 to 30 June 2010 in rural and metropolitan hospitals in South Australia, representing all independent contiguous cases of MI in South Australia during that time period.	**Aim**<br>• To evaluate the impact of the regionalised Integrated Cardiovascular Clinical Network (ICCNet) on 30-day mortality among patients with acute myocardial infarction (MI) presenting to hospitals in a rural setting.<br>**Intervention**<br>Three key design features of the network:<br>• Standardised risk stratification and evidence-based treatment protocols<br>• Point-of-care testing for whole-blood troponin T levels with central quality control<br>• A designated on-call consultant cardiologist to ensure response within 10 minutes and facilitation of transfer to metropolitan hospitals<br>**Design**<br>Retrospective state-wide observational comparison of outcomes before and after the establishment of a regionalised integrated Cardiovascular clinical network<br>**Method**<br>• Analysis of routinely	• The mean predicted 30-day mortality was lower among rural patients compared with metropolitan patients, while actual mortality rates were higher (30-day mortality: rural, 705/5630 [12.52%] v metropolitan, 2140/23 993 [8.92%]; adjusted odds ratio [OR], 1.46; 95% CI, 1.33–1.60; *P*< 0.001).<br>• Overall, annual mortality rates declined over the 9 years (per year, $OR_{risk\text{-}adj}$ 0.97 [95% CI, 0.95–0.99]; *P* < 0.001). However, these declines were greater in rural areas (interaction between year and rural location, *P* = 0.04). In 2001, the adjusted OR for patients presenting in rural areas was 1.69 (95% CI, 1.40–2.04; *P* < 0.001), but by 2010 this was no longer significant.<br>• Among rural hospitals, 30-day mortality was lower among patients presenting to hospitals integrated into the clinical network compared with those not in the network (OR=0.78; P=0.007).<br>• After adjustment for temporal improvement in MI outcome, baseline comorbidities and MI characteristics, availability of immediate cardiac support (i.e. presentation to an ICCNet hospital) was associated with a 22% relative odds reduction in 30-day mortality (OR, 0.78; 95% CI, 0.65–0.93; *P*= 0.007).<br>• A strong association between network support and transfer of patients to metropolitan hospitals was observed (before ICCNet, 1102/2419 [45.56%] v after ICCNet, 2100/3211 [65.4%]; *P*< 0.001). Increased transfers were associated with a lower total length of stay compared with

Reference	Country	Type of Network	Sample	Study Aim, Design, Method and Indicators	Primary Results
				collected data for patients with a diagnosis of myocardial infarction pre- and post-implementation of the network, comparing rural network hospitals with rural non-network hospitals and metropolitan hospitals.<br>**Indicators**<br>• Risk-adjusted 30-day mortality<br>• Rate of transfer of rural patients to metropolitan hospitals<br>• Proportion of patients receiving angiography	admissions before implementation of the network.<br>• Rates of angiography increased among rural patients, but remained lower than metro patients. The difference between rural and metro patients diminished over the time period.<br>• Increasing co-morbidities were associated with a lower likelihood of transfer among rural patients. Patients presenting to rural hospitals within the network were more likely to be transferred to a metro hospital than patients presenting to rural hospitals outside the network (OR=2.23; P<0.001) and were associated with a reduction in mortality across all degrees of comorbid risk.

Qualitative Articles

Reference	Country	Type of Network	Sample	Study Aim, Design, Method and Indicators	Results
▪ **Features and outcomes of effective networks**					
Ahgren & Axelsson. 2007	Sweden	'Chains of care' (managed clinical networks) for patients having the same illness or symptom	6 chains of care networks – 3 selected to be successful<br><br>3 selected to be unsuccessful in developing chains of care in 4 counties.	**Aim**<br>To identify the factors and their relative importance that may be important for the development of chains of care<br>**Design**<br>Cross-sectional embedded multiple-case study<br>**Method**<br>Semi-structured group and individual interviews and studies of documents<br>**Indicators**<br>*Success of network:*<br>Extent of functional integration that included clinical, administrative as well as financial integration within the chain of care.<br>*Explanatory factors were:*<br>▪ Development focus<br>▪ Development opportunities<br>▪ Organisational structure<br>▪ Organisational culture<br>Each sub-unit of analysis had several indicators.	▪ Success of networks was based on the extent of their functional integration<br>▪ It was important that the focus of the development was compatible with the culture of the organisations<br>▪ 3 networks were considered to be unsuccessful based on their lack of functional integration<br>▪ The three major determinants of successful networks were: professional dedication of the staff within the networks; legitimacy of the network; confidence of the staff and organisations involved.<br>▪ Networks initiated locally by dedicated professionals, physicians in particular, are more likely to have a successful outcome
Baker & Wright 2006	UK	Managed clinical network for paediatric liver services	93 practitioners, patients, families of patients, drug company representatives	**Aim**<br>To address the special problems arising from tension between need for centralisation of skills and advantages of decentralisation of care	▪ The requirements of patients and families overlapped with the ideals of professionals<br>▪ Results of the three sessions agreed broadly on the elements essential to the creation of a successful clinical network<br>▪ Key elements included patient education, open

Reference	Country	Type of Network	Sample	Study Aim, Design, Method and Indicators	Results
			and NHS managers	**Design/Method**<br>Appreciative enquiry sessions held in 3 locations: Crieff, Birmingham & London<br>**Indicators**<br>▪ Learning<br>▪ Roles and relationships<br>▪ Share-care and liver disease at home<br>▪ Access to services/first interaction<br>▪ Standards, protocol and safety	and inclusive communication, customer care, a clear care pathway, and national protocols, guides and standards<br>▪ The vision for the MCN was partner relationships, respect for autonomy, personal autonomy, information and service access, least possible disruption of normal life with flexibility according to personal needs and patient centredness<br>▪ Features of a successful network were identified as:<br>▪ Care as close to home as possible<br>▪ Open and inclusive communication<br>▪ A clear care pathway<br>▪ Better customer care including interactions with a key worker/coordinator
Cunningham et al 2012	Australia	Advisory clinical networks – two networks for musculoskeletal health in two states in Australia (New South Wales and Western Australia)	36 interviews with key informants (network managers, network members and stakeholders including representatives from Departments of Health and clinical and non-governmental organisations)	**Aim**<br>To describe the features and roles of clinical networks and identify factors relating to clinical network effectiveness and sustainability, and to explore achievements of the networks.<br>**Design**<br>Longitudinal comparative case study<br>**Methods**<br>Semi-structured in-depth interviews to ascertain perceptions of network members and stakeholders regarding key factors relating to clinical network effectiveness and sustainability conducted between March-August 2011<br>• 19 of 92 core members in	• Interviewees perceived a network to be successful:<br>▪ At the community level if there was greater consultation, greater agreement and acceptance of network recommendations, greater implementation of Models of Care, improving practice patient care and measureable improvement in patient outcomes;<br>▪ At the network level if the network was able to get together measured by growth in network membership, broad stakeholder representation, and contribution of the network manager and network leadership;<br>▪ At the member level if there is member participation and responsiveness in the network, member contribution to the network, and success in embedding practice changes in the member's own hospital/clinic.<br>▪ Network manager and leadership were perceived

Reference	Country	Type of Network	Sample	Study Aim, Design, Method and Indicators	Results
				NSW interviewed<br>• 17 of 34 core members in WA interviewed<br>**Indicators**<br>• Measures of effectiveness at the community, network and member level at the short, medium and long term<br>• Key achievements of each network	as being critical for the success of the networks.<br>▪ Both networks used a distributive leadership model, and a structure of establishing key working groups led by expert members of the network.<br>▪ Stakeholders noted the role of networks in identifying gaps between current practice and evidence-based practice; directing care into more evidence-based practices and improve professional/patient interface; collaboration across health sites; effective communication with and inclusion of a broad range of stakeholders; engaging clinicians and enabling them to contribute to policy.<br>▪ Challenges included funding and a disconnection between network recommendations and implementation especially if the network did not have the authority for implementation.
Hogard & Ellis 2010	UK	Managed clinical network for personality disorder (PD)	All members of staff involved in the MCN	**Aim**<br>To evaluate how the network had performed in its purpose to establish a better coordinated service for patients with PD and what changes or refinements might be required<br>**Design**<br>Evaluation Trident methodology<br>**Method**<br>Evaluation of outcomes, processes and multiple stakeholder perspectives over a 2 year period including: interviews, focus groups, telephone interviews, questionnaires, documentation analysis and NHS data sets. Processes were further	▪ On the basis of the audit, staff in the network could be described as in a partnership in that they shared values and objectives. However such commitments in principle do not guarantee clinical effectiveness.<br>▪ Positives of the network reported included being able to provide a holistic service to users including provision of a nonmedical assessment and formulation and ultimately encouraging better engagement with clients. The wide range of services linking into the network was also commended.<br>▪ Negatives of the network reported included a lack of funding and resources leading to limited capacity to coordinate care for a large number of clients, the speed with which the network was able to process referrals, and poor communication. Tension in relationships between

Reference	Country	Type of Network	Sample	Study Aim, Design, Method and Indicators	Results
				assessed using the following standardised dedicated measures: A partnership audit tool (PAT); a care programme approach audit tool (CPA) and the PD self-capabilities framework self-audit tool (PDCF).<br>**Indicators:**<br>▪ *Outcomes*- Focus on 2 key outcomes relating to effectiveness of treatment provided: reduction in frequency of crises; reduction in inappropriate service use<br>▪ *Process* – 5 main focuses: organisational and functional structure; service user pathway; partnership; care planning approach (CPA); staff development needs<br>▪ *Stakeholder interviews* – explored five core themes: 1. Attitude prior to joining the network; 2. Attitude changes as a result of joining the network; 3. The impact of MCNs; 4. Working relationships; and 5. The value added by the PD MCN	network staff and referrers were also reported, with participants noting a need to improve working relationships and transfer of knowledge.<br>▪ Record keeping for assessment and clinical assessment was at an early stage and there was a need for a more systematic use of assessment instruments and data management instruments<br>▪ The service did not keep appropriate information that could be used to measure outcomes and tools to measure crisis were being used inconsistently by network staff. There were challenges in capturing whether there was an impact for service users and a lack of evidence regarding clinical outcomes.<br>▪ Much of what was reported in this evaluation relied on anecdotal data, due to a lack of formal evidence.<br>▪ While the network had achieved its objectives to establish new operational structures it was unclear whether it had maintained or improved clinical services.<br>▪ Stakeholder interviews indicated that prior to joining the MCN a number of staff had previously viewed PD in a negative light. Many staff reported that their attitude towards PD had not changed since joining the network but a number did explain that their knowledge and experience had increased significantly.<br>▪ Staff highlighted the benefits of working as part of a MCN which was viewed as a way to provide an efficient and informed service.<br>▪ Working relationships within the MCN were viewed positively on the whole, despite some tensions between network staff and the referrers.<br>▪ The MCN was considered by staff to have added value by raising the profile of PD and helping to

Reference	Country	Type of Network	Sample	Study Aim, Design, Method and Indicators	Results
					share skills and knowledge across a number of agencies and services.<br>▪ However the benefits of the MCN "remain theoretical rather than proven".
McInnes et al 2012	Australia	Voluntary collegial clinical networks in New South Wales, Australia established by the NSW Agency for Clinical Innovation	27 interviews with network drivers especially network managers (9), network participants (6), senior health service managers in a clinical operations or clinical governance role at a hospital (4), and senior policy-makers (8).	**Aim**<br>To identify key stakeholders' views on the conditions required to establish successful and effective clinical networks and what they identify as outcomes of successful clinical networks.<br>**Design**<br>Comparative case study<br>**Methods**<br>A purposive maximum variation sampling approach was used to recruit the four types of participants. 27 individual semi-structured face-to-face interviews were conducted. Sample size was determined by saturation of themes.<br>**Indicators**<br>• Factors necessary for effective networks<br>• Outcomes indicating whether clinical networks are effective	▪ Factors necessary for networks to be effective included:<br>▪ Building relationships within and with external networks and a strong commitment to the networks<br>▪ A bottom-up approach to integration, preferably locally-initiated but with formalisation of the networks<br>▪ Supportive policy environments and links with state health agencies and local health services<br>▪ Strong leadership, including passionate clinical leaders, was necessary for effective structure, organisation and governance<br>▪ A strategic, feasible evidence-based work plan with measureable milestones and that was valuable to participants<br>▪ Adequate resources including a dedicated network manager and technological resources<br>▪ The ability to implement changes in practice or service delivery to address gaps in current practice, that are relevant to members, feasible and measureable<br>▪ Features of ineffective networks included:<br>▪ Lack of funding and resources<br>▪ Tension between network members<br>▪ Poor communication<br>▪ Poor record keeping making it difficult to assess impact<br>▪ Poor teamwork and working relationships<br>▪ Lack of inclusion of certain populations

Reference	Country	Type of Network	Sample	Study Aim, Design, Method and Indicators	Results
					▪ The following outcomes of successful clinical networks were identified:<br>▪ Better working relationships and greater interdisciplinary collaboration in patient care and development of research projects<br>▪ Open and transparent partnerships with external stakeholders such as the health department and greater mutual understanding of perspectives<br>▪ More effective clinical services reflected by improving patient journeys, clear care pathways, provision of holistic services, standardising care, reducing variation in care, reducing costs and monitoring quality<br>▪ Implementation and wide-scale spread of network initiatives and impact on practice<br>▪ Growth of the network
▪ **Network Implementation**					
Fleury et al 2002	Canada	Mental health integrated service network	N = 143 staff and administrators at all levels of service intervention, clients of self-help groups and outpatient clinics and relatives and friends of the mentally ill selected using an intentional sampling strategy and interviewed in	**Aim**<br>To examine the process of implementing regional planning and the influence of contextual, structural, cultural and dynamic factors on forming networks<br>**Design**<br>Case study and multi-dimensional analytic model<br>**Method**<br>• Interviews<br>• Review of primary sources (e.g. minutes, correspondence, administrative documents and policies)<br>• Review of secondary sources	▪ The study found that regional planning involving stakeholders was not sufficient for implementing mental health care networks integration as it did not create a genuine reconfiguration of services<br>▪ Successful implementation was inhibited by several factors including:<br>▪ the large number of professionals involved in different services,<br>▪ ambivalence towards network priorities when and if opposed to organisational priorities and rigidity of established practices,<br>▪ centrality rather than dispersion of power,<br>▪ the lack of recognition of legitimacy and expertise of planners,<br>▪ irreconcilable visions of system structuring,<br>▪ the lack of clinical, function and professional integration,<br>▪ hospitals maintained a centralised position in

Reference	Country	Type of Network	Sample	Study Aim, Design, Method and Indicators	Results
			six time periods from Winter 1995 to Summer 1997	(e.g. mental health, organisational theory, network literature)<br>**Indicators**<br>The framework focused on three lines of analysis:<br>• Context for implementing the regional planning procedure<br>• Determinants of implementing and impact of regional planning such as problem-setting, network direction setting and structuring<br>• The dynamic of developing regional planning	the networks that allowed them to hoard resources.<br>▪ The study reinforced that reform can only be implemented with the approval and genuine participation of the professionals directly involved the field
Tolson et al 2005[9]	Scotland	Managed clinical network (Palliative Care), linking primary, secondary and tertiary care	1 network sample in study<br><br>3 older men, their families, the doctors and nurses providing care, along with 13 members of the network management group	**Aim**<br>▪ To evaluate, refine and inform the ongoing development of the MCN.<br>▪ To reflect of the merits and challenges of a realistic evaluation design in establishing a new palliative care MCN to implement a care guideline for pain management in a primary care setting.<br>**Design**<br>A "realistic evaluation design". A qualitative pilot study evaluating guideline implementation at three separate points (6, 11, and 15 months) during the implementation of managed	▪ Progress in establishing the network was much slower than expected and was hindered by: inexperience in change management and unfamiliarity with leading practice development projects and supporting practitioner learning.<br>▪ Co-ordination, leadership and strategic support (particularly professional buy-in) in change-management were critical to success.<br>▪ There was a consistent trend of an increasing recognition over time about the pivotal role of practitioners in the development of the network.<br>▪ Professional outcomes centred on improved team working and enhanced communication, increased knowledge, greater satisfaction, reflective practice and increased commitment to evidence-based care.<br>▪ In terms of patient outcomes, there was accumulating evidence of better pain management and symptom control, and increased

Reference	Country	Type of Network	Sample	Study Aim, Design, Method and Indicators	Results
				clinical network.<br>**Method**<br>Findings from qualitative interviews and patient-level clinical data comprised case studies of patient-centred experiences of care. These case studies, along with semi-structured interviews with health-care professionals informed the evaluation, reviewed and refined by the network executive.<br>**Indicators**<br>Relationships between:<br>▪ Context<br>▪ Mechanisms<br>▪ Outcomes	knowledge through better patient education.<br>▪ The amount of time and input demanded of the coordinator role was high and increased with wider reach of the network.<br>▪ The length of time and effort required to achieve buy-in into the network is high. This often involves a “values reconciliation” phase where members examine and compare their own/team values and practices to those of the network.<br>▪ Networks can only be effective if the appropriate (often numerous) steps are taken and the context is favourable (e.g. clinicians are receptive to audit and feedback).
Touati et al 2006[8]	Canada	Managed clinical network (cancer)	5 hospitals offering oncological services in the Quebec region	**Aim**<br>To determine the extent of clinical leadership as a means for transforming health care in an oncological services network<br>**Design**<br>Longitudinal qualitative case study using process analysis to examine how the networks influenced change<br>**Method**<br>Data collected from 1999-2003 included:<br>▪ Non-participant observation of 50 administrative meetings relating to governance of change<br>▪ 65 semi-structured interviews with network promoters	▪ Inter-professional and inter-organisational trust developed in all hospitals. However the level of commitment by physicians and professionals to the implementation of the network varied.<br>▪ All of the hospitals attempted to stabilise oncology teams and felt that they benefited from administrative support to set up clinical teams.<br>▪ In varying degrees all hospitals implemented measures to foster cooperation between professionals. Interdisciplinary team meetings were being held in 4 out of 5 hospitals but oncologists did not participate in all hospitals.<br>▪ In 4 out of 5 hospitals, most respondents shared the philosophy and vision promoted by the governance of the network with regard to: response to all of the individual’s needs; coordinated care; standardisation of clinical practices; and patient-centered care.<br>▪ Clinical leadership is effective in implementing

Reference	Country	Type of Network	Sample	Study Aim, Design, Method and Indicators	Results
				including clinician leaders, professionals from multidisciplinary teams, hospital managers<br>▪ Document analysis (e.g. protocols, budget statements)<br>**Indicators**<br>▪ Origins of change<br>▪ Facets of integration:<br>- normative<br>- functional<br>- clinical	change but is limited. Contextual variables, the nature of the changes emphasized (those consistent with the actors' values and interests) fostered change.<br>▪ Positive change is more likely to be achieved by a 'constellation of clinical, administrative and political leaders' at different levels of the health care system.<br>▪ To enhance the coordination of care, coordination committees were set up to jointly formalise processes involving nursing care case-management. Longstanding collaboration facilitated the implementation of these committees.<br>▪ The study highlights the complexity of health services integration processes which demand considerable time, resources and initiatives at different levels of the health system.
▪ **Organisational Structure**					
Addicott R 2008	UK	Managed clinical network for cancer services	117 professionals from 5 cancer networks in London	**Aim**<br>To explore the changing model of governance in the UK, particularly the increasing focus on networks and the role of the network Board<br>**Design**<br>Comparative case study<br>**Method**<br>▪ Semi-structured interviews with nurses, clinicians, managers and policy makers<br>▪ Document analysis<br>▪ Observation at meetings<br>**Indicators**<br>▪ Network structure<br>▪ Purpose of the network	▪ Cancer network management teams and Boards had limited strategic influence as networks were constrained by a continued emphasis on centralised performance management and structural reconfiguration<br>▪ Success of decision making was dependent on seniority of representation on the network Board. In only 1 out 5 networks the Board had high representation from extremely senior representatives and this Board had a noteworthy impact on strategic decision making.<br>▪ Both the network management teams and Board only had minimal decision-making influence within a prevailing centralised bureaucratic structure. Although the espoused logic of the network was to decentralise decision making to a local level, power and budgetary responsibilities

Reference	Country	Type of Network	Sample	Study Aim, Design, Method and Indicators	Results
				▪ Network Management Team approach to networking<br>▪ Characteristics of the Board<br>▪ Approach to organisational change	ultimately remained centralised. Network Boards have had limited scope for strategic decision making.<br>▪ The key finding is that the managed network model was not powerful enough to deinstitutionalise the prevailing governance discourse of performance management and centralised accountability.
Addicott R & Ferlie E 2007	UK	Managed clinical network for cancer services	117 professionals from 5 cancer networks in London	**Aim**<br>To explore and theorise the nature of power relations within a network model of governance<br>**Design**<br>Comparative case study<br>**Method**<br>▪ Semi-structured interviews with nurses, clinicians, managers and policy makers<br>▪ Document analysis<br>▪ Observation at meetings<br>**Indicators**<br>3 tracers of power relationships:<br>▪ Centralisation of specialist services<br>▪ Budget/resource allocation<br>▪ Education and training activities	▪ The 5 networks were structured in similar ways due to the national policy agenda.<br>▪ Network Management Teams had no statutory influence or performance management mechanism and had to rely on interpersonal skills to influence cooperation. A lack of these skills frequently resulted in inability to generate meaningful changes or control the delivery of services.<br>▪ Decision making was dominated by medical staff in all 5 networks.<br>▪ During localised decision-making and implementation of policy less dominant medical professionals presented barriers in an attempt to exert influence.<br>▪ These cases demonstrated that the internal divisions in the medical profession, with active power and influence unevenly distributed in favour of those in the cancer centre while less powerful medical professionals were then forced into defensive mode to resist decisions that had been made.
Addicott R, McGivern G & Ferlie E 2007	UK	Managed clinical network for cancer services	117 professionals from 5 cancer networks in London	**Aim**<br>To explore how stakeholders involved in the delivery of cancer services in the UK adopted or adapted managed clinical networks as a novel managerial	▪ The knowledge sharing purpose of networks was distorted by top-down structural reorganisation demands of central government resulting in superficial bottom-up adoption of the networks models and a lack of focus on process or strategic issues.

Reference	Country	Type of Network	Sample	Study Aim, Design, Method and Indicators	Results
				technique for sharing best practice and knowledge<br>**Design**<br>Comparative case study<br>**Method**<br>▪ Semi-structured interviews with nurses, clinicians, managers and policy makers<br>▪ Document analysis<br>▪ Observation at meetings<br>**Indicators**<br>3 tracers of knowledge management:<br>▪ Centralisation of specialist services<br>▪ Budget/resource allocation<br>▪ Education and training activities (an indicator for knowledge management activity)	▪ The centralisation process was feared by clinicians and negatively impacted on alternative educational and knowledge sharing activities.<br>▪ In 4 out of 5 networks there was frequent resistance to making decisions and implementing changes.<br>▪ One network demonstrated greater network-wide investment in education and training activities. This was largely due to a strong, well-perceived Network Management Team which began to develop an educational strategy across the network.<br>▪ Overall, networks had little impact on organisational processes. The majority of networks had a limited focus on educational and training activities, and broader issues surrounding organisational change.<br>▪ One network was an outlier. An open and facilitative approach to managing networks was more successful. The network was more successful in building on pre-existing relationships that were evident prior to establishment of the networks. Those involved in managing and leading the network were successful in considering the needs of the local context during the process of implementing the network.
▪ **Organisational Learning and Knowledge**					
Addicott et al 2006	UK	Managed clinical network for cancer services	117 professionals from 5 cancer networks in London	**Aim**<br>To explore whether the knowledge management function of managed clinical networks was realised in practice<br>**Design**<br>Observational, cross-sectional organisational process study	▪ There was little evidence of change in practice within 4 out of 5 networks. This was considered to be a result of interorganisational competition following from structural reconfiguration, an emphasis on achieving targets and conformance with protocols and persistent interprofessional boundaries.<br>▪ In 1 out of 5 networks there was cohesion within

Reference	Country	Type of Network	Sample	Study Aim, Design, Method and Indicators	Results
				**Method**<br>▪ Semi-structured interviews with nurses, clinicians, managers and policy makers<br>▪ Document analysis<br>▪ Observation at meetings<br>**Indicators**<br>Network impact on:<br>▪ structural reconfiguration<br>▪ budgetary allocation<br>▪ educational and training activity	the network and the structural reconfiguration process resulted in significant changes in practice.<br>▪ In this 'successful' network, there was more evidence of learning, training, knowledge sharing, and education. This was thought to be due in part to the network being well and supportively managed, facilitating engagement, having a detailed understanding of cancer services, a localised appreciation for the dynamics of the organisations involved, and good pre-existing relationships between members of the network prior to commencement.<br>▪ Lack of success in the other four networks was perceived as being due to limited time and resources, lack of enthusiasm from network members, and increased competition for resources within each network. Respondents from cancer centres were more positive about the learning aspects of the networks than representatives from peripheral units. Some thought that learning would become a greater priority when structural reconfigurations were underway or complete.
Burnett et al 2005	UK	Managed clinical networks (MCNs)	9 interviewees from Scottish MCN priority areas: cancer, coronary heart disease, stroke and mental health and a representative from local health community co-	**Aim**<br>To explore the extent to which the information culture and practices within MCNs and whether they are able to deliver improved care<br>**Design/Method**<br>Qualitative information and knowledge needs analysis comparing responses from MCN respondents with those from a previous study of staff working in a more traditional environment	▪ Evidence-based practice was a requirement within the Scottish Health Service in general and within the MCN in particular, noting the importance of being able to access information.<br>▪ Individuals working within the MCN perceived that information and knowledge had an impact on service delivery and demonstrated a greater ability to reflect on the value of knowledge and information in their roles<br>▪ Information and communication technologies (and in particular the e-Library) was widely recognised as an important for access to health

Reference	Country	Type of Network	Sample	Study Aim, Design, Method and Indicators	Results
			operative; respondents represented a range of roles including specialist nurses, lead clinicians, planning and implementation managers.	**Method**<br>• Semi-structured in-depth interviews; approximately 1 hour in duration.<br>**Indicators**<br>• How MCN staff used knowledge in their roles, requirements of the knowledge base and problems with knowledge provision;<br>• Role of information in supporting evidence based practice<br>• Perceptions of the e-Library<br>• Education and training<br>• IT support<br>• Barriers to the use of information	care knowledge and MCN respondents reported a greater need for and confidence in information literacy.<br>▪ MCN respondents also considered colleagues an important source of information with emphasis on the inter-disciplinary and cross-boundary aspects of MCNs facilitating knowledge transfer.<br>▪ Healthcare professionals in MCNs discussed information facilitating communication with patients and including patients as a part of the "knowledge network".<br>▪ The MCN group demonstrated an ability to reflect on the value of information and knowledge in their roles. They saw information and knowledge as having an impact on service delivery. They also recognised that it is vital to have easy and timely access to the information and knowledge they require to operate as effectively and efficiently as possible.
